# Three-dimensional environment sensitizes pancreatic cancer cells to the anti-proliferative effect of budesonide by reprogramming energy metabolism

**DOI:** 10.1186/s13046-024-03072-1

**Published:** 2024-06-14

**Authors:** Eduardo Ibello, Federica Saracino, Donatella Delle Cave, Silvia Buonaiuto, Filomena Amoroso, Gennaro Andolfi, Marco Corona, Ombretta Guardiola, Vincenza Colonna, Bruno Sainz Jr, Lucia Altucci, Dario De Cesare, Gilda Cobellis, Enza Lonardo, Eduardo Jorge Patriarca, Cristina D’Aniello, Gabriella Minchiotti

**Affiliations:** 1 Institute of Genetics and Biophysics, ’A. Buzzati-Traverso’, CNR, Naples, Italy; 2 Department of Precision Medicine, University of Campania Luigi Vanvitelli, Naples, Italy; 3 Department of Genetics, Genomics and Informatics, University of Tennessee Health Science Center, Memphis, TN USA; 4 Department of Cancer, Instituto de Investigaciones Biomedicas Sols-Morreale (IIBM), CSIC- UAM, Madrid, 28029 Spain; 5 Cancer, Area 3-Instituto Ramon Y Cajal de Investigacion Sanitaria (IRYCIS), Madrid, 28034 Spain; 6 Centro de Investigación Biomédica en Red, Área Cáncer, CIBERONC, ISCIII, Madrid, 28029 Spain; 7 BIOGEM, Ariano Irpino, Ariano Irpino, AV 83031 Italy; 8 IEOS-CNR, Naples, 80100 Italy; 9 Medical Epigenetics Program, AOU Vanvitelli, Naples, Italy

**Keywords:** Pancreatic cancer, 3D spheroids, Metabolic reprogramming, Cell proliferation, Budesonide, Glucocorticoids

## Abstract

**Background:**

Pancreatic ductal adenocarcinoma (PDAC) is the most lethal cancer with an aggressive metastatic phenotype and very poor clinical prognosis. Interestingly, a lower occurrence of PDAC has been described in individuals with severe and long-standing asthma. Here we explored the potential link between PDAC and the glucocorticoid (GC) budesonide, a first-line therapy to treat asthma.

**Methods:**

We tested the effect of budesonide and the classical GCs on the morphology, proliferation, migration and invasiveness of patient-derived PDAC cells and pancreatic cancer cell lines, using 2D and 3D cultures in vitro. Furthermore, a xenograft model was used to investigate the effect of budesonide on PDAC tumor growth in vivo. Finally, we combined genome-wide transcriptome analysis with genetic and pharmacological approaches to explore the mechanisms underlying budesonide activities in the different environmental conditions.

**Results:**

We found that in 2D culture settings, high micromolar concentrations of budesonide reduced the mesenchymal invasive/migrating features of PDAC cells, without affecting proliferation or survival. This activity was specific and independent of the Glucocorticoid Receptor (GR). Conversely, in a more physiological 3D environment, low nanomolar concentrations of budesonide strongly reduced PDAC cell proliferation in a GR-dependent manner. Accordingly, we found that budesonide reduced PDAC tumor growth in vivo. Mechanistically, we demonstrated that the 3D environment drives the cells towards a general metabolic reprogramming involving protein, lipid, and energy metabolism (e.g., increased glycolysis dependency). This metabolic change sensitizes PDAC cells to the anti-proliferative effect of budesonide, which instead induces opposite changes (e.g., increased mitochondrial oxidative phosphorylation). Finally, we provide evidence that budesonide inhibits PDAC growth, at least in part, through the tumor suppressor CDKN1C/p57Kip2.

**Conclusions:**

Collectively, our study reveals that the microenvironment influences the susceptibility of PDAC cells to GCs and provides unprecedented evidence for the anti-proliferative activity of budesonide on PDAC cells in 3D conditions, in vitro and in vivo. Our findings may explain, at least in part, the reason for the lower occurrence of pancreatic cancer in asthmatic patients and suggest a potential suitability of budesonide for clinical trials as a therapeutic approach to fight pancreatic cancer.

**Supplementary Information:**

The online version contains supplementary material available at 10.1186/s13046-024-03072-1.

## Background

Pancreatic ductal adenocarcinoma (PDAC), the most frequent type of pancreatic cancer (PC), is a highly aggressive solid tumor with poor clinical outcome [1]. Incidence and mortality are increasing every year, and PC is predicted to become the second leading cause of cancer-related death by 2030 [2, 3]. These poor statistics are due to a lack of screening methods, late diagnosis, and limited response to chemo- and radiotherapy, which makes PC a priority for the development of novel and more efficient therapies. Despite increasing efforts to improve PDAC management, none of the therapies developed so far have been proven to be clinically curative. Interestingly, recent findings revealed the existence of an inverse association between chronic asthma and PDAC occurrence, even though the mechanism underlying this correlation is still unknown [4]. Asthma is an inflammatory disease, whose main treatment relies on the use of anti-inflammatory drugs, such as glucocorticoids (GCs). Emerging evidence suggests that chronic inflammation may also play a role in promoting pancreas carcinogenesis [5], thus raising the hypothesis that a GC-dependent inhibition of the immune cell response may explain, at least in part, the negative association between asthma and PDAC. A first-line therapy to treat asthma is budesonide [6, 7], a hydrophobic GC with an extensive first-pass hepatic metabolism. Thus, budesonide has low systemic bioavailability and minimal side effects, such as the suppression of the hypothalamic-pituitary-adrenal axis [8–10]. Of note, beside the well-known anti-inflammatory effect of budesonide, previously unrecognized activities of this drug have been recently reported. Indeed, budesonide and its analogues induce morphological and behavioral changes in stem and cancer cells [11–13]. Specifically, budesonide hinders exit from naïve pluripotency and acquisition of mesenchymal traits in mouse embryonic stem cells (ESCs), and prevents ESC aggregates to break symmetry and develop into embryo-like structures named 3D gastruloids [11–13]. Moreover, budesonide reduces mesenchymal traits and induces epithelial-like features in lung and triple negative breast cancer cells, in vitro and in vivo [13]. Based on these recent findings and the above considerations, here we aimed to explore a potential link between budesonide therapy and the reduced incidence of PC in asthmatic patients.

We investigated the effect of budesonide on PDAC tumor cell behavior in vitro and in vivo. Specifically, we revealed a previously unrecognized effect of budesonide on the invasive features of PDAC cells in 2D culture conditions. Indeed, we demonstrate that a high (µM) budesonide regimen reduces the mesenchymal and invasive characteristics of PDAC cells, without affecting their proliferation. Unexpectedly, under 3D culture conditions (i.e. floating tumor spheroids and organotypic cultures), in the same growth medium of the 2D setting, budesonide inhibits PDAC cell proliferation already at low nanomolar (nM) concentrations. In line with these findings, budesonide significantly reduced PDAC tumor growth in vivo. The anti-proliferative effect of budesonide is glucocorticoid receptor (GR)-dependent and relies on the expression of the GR target gene cyclin dependent kinase inhibitor 1 C (*CDKN1C*) [14]. Mechanistically, budesonide drives GR-dependent metabolic changes, which contrast with the metabolic profile required to proliferate under 3D conditions. Our results suggest that the response of PDAC cells to GCs strictly relies on the growth environment.

## Methods

### Cell lines, culture conditions and treatments

The human pancreatic cancer cell line PANC1 was purchased from ATCC (CRL-1469™); tumor-derived PDAC#253 and #354 cell lines were previously isolated and characterized from two independent PDAC patients [15, 16]; L3.6pl cell line was previously selected as a metastatic variant of PDAC [17]. All cell lines were routinely tested for Mycoplasma-free state and used within passage 28.

Patient-derived PDAC cell lines were cultured in PDAC growth medium: RPMI 1640 Medium, GlutaMAX™ Supplement (Invitrogen), 10% FBS South America (Euroclone), Penicillin/Streptomycin (Invitrogen), Normocin (InvivoGen). L3.6pl and PANC1 were cultured in DMEM (Invitrogen), 10% FBS (Euroclone), Penicillin/Streptomycin and L-Glutamine (Invitrogen).

Two-dimensional (2D) assays, including proliferation, colony morphology, migration, and Cy3-gelatin invadopodia assays, were performed starting from PDAC/PANC1 cells grown/maintained at low culture density (prevalence of cell-substrate adhesive contacts) by performing at least 6 passages at a low 1:6 ratio. For drug treatments PDAC#253 and #354 cell lines were plated (1.5 × 10^4^ cells/cm^2^) at day − 1 on gelatin-coated plates. At day 0, cells were treated ± budesonide (2.5 to 20 µM), dexamethasone (20 µM), or DMSO for three days. PANC1 (5 × 10^3^ cells/cm^2^) were plated on gelatin-coated plates ± budesonide (20 µM), dexamethasone (20 µM) or vehicle DMSO for four days, with medium refresh ± drugs, at day 2. Upon the treatments, both PDAC and PANC1 cell lines were either fixed/stained with a solution of 1X PBS/6% glutaraldehyde/0.15% crystal violet or used for other applications (see below).

Budesonide and hydrocortisone were dissolved in DMSO, while dexamethasone (Sigma-Aldrich) was dissolved in H_2_O at 10 mM.

### Generation of *NR3C1* KD PDAC cells

For the generation of *NR3C1* KD cells, PDAC#253 cells were infected with lentiviral particles carrying a short hairpin RNAs (shRNA) targeting *NR3C1* gene and a resistance to puromycin [18]. An empty vector (shEmpty) was used as a control. After 2 days in culture, cells were subjected to puromycin selection (5 µg/ml) for 7 days.

### Generation of *CDKN1C* KD PDAC cells

For the generation of *CDKN1C* KD cells, PDAC#253 cells were nucleofected with siRNA targeting *CDKN1C* gene (Thermo Fisher). A non-targeting siRNA was used as a control.

### 2D cell proliferation and cell apoptosis assays

To evaluate cell proliferation and apoptosis, PDAC#253, #354 and PANC1 cell lines were treated as described above and subjected to either 5-ethynyl-2’-deoxyuridine (EdU; Click-iT EdU Flow Cytometry Assay Kit, Invitrogen) proliferation or Annexin V/Propidium iodide staining (Dojindo Laboratories) apoptosis assays. Briefly, for cell proliferation, cells were incubated overnight with EdU (10 µM), dissociated, fixed and stained following manufacturer’s instructions. Samples were analyzed by flow cytometry (FACS-ARIAIII, Becton-Dickinson). For cell apoptosis, samples were analyzed with a FACS-Canto using the DivaTM software (BD Biosciences).

### 2D migration assays

Boyden chamber assays were performed using polycarbonate transwells (8 μm-pore, Costar). PDAC#253 and #354 cell lines were treated for three days as described above ± budesonide (20 µM) or DMSO in complete medium. After 3 days, cells were dissociated with trypsin-EDTA (Invitrogen) and seeded (2.5 × 10^4^ cells/well) in RPMI/1% FBS ± budesonide (20 µM) and allowed to migrate towards FBS gradient (from 1 to 10%) for 18 h. Cells were fixed/stained with a solution of 1X PBS/6% glutaraldehyde/0.15% crystal violet and migrated cells were counted (8 fields/well) using ImageJ software.

### 3D organotypic culture

3D organotypic culture was performed as previously described [13]. Briefly, PDAC#253, #354 and PANC1 (1 × 10^3^ cells/well) were plated in 8-well chamber slides, onto a base layer of 100% matrigel and supplemented with 2% matrigel/PDAC growth medium (RPMI + 10% FBS) ± budesonide (20 µM) for 6 days. 3D spheroid area and morphology (cohesive/spherical *versus* not cohesive/invasive spheroids) were analyzed using ImageJ software. PKH26 (SIGMA) staining was performed by staining PDAC#253 cells in suspension with the dye (1:1) before plating onto matrigel, as described above.

### Cy3-Invadopodia assay

The Cy3-gelatin Invadopodia assay (Millipore) was performed by plating budesonide, dexamethasone- and DMSO-treated PDAC#253, #354 and PANC1 cells (1 × 10^5^ cells/cm^2^) onto 8-well chamber slides coated with the Cy3-gelatin, following the manufacturer’s instructions. Cells were fixed with 4% PFA and stained with phalloidin (FITC) and DAPI. The presence of areas of degraded Cy3-gelatin in correspondence of the cells was visualized by confocal microscopy and analyzed by ImageJ. Degraded area (pixel/cm^2^) was measured over the total number of nuclei.

### Spheroid formation assay

Three-dimensional (3D) assays were performed using cells adapted to a high-density culture condition, by performing at least 6 passages at a high 1:2 ratio. These high-density passages improved the ability of pancreatic cancer cells (PDAC, PANC1 and L3.6pl cells) to aggregate, generating strong cell-cell adhesive contacts, and thus reproducible floating cell aggregates. Tumor spheroid assays were performed by plating 5 × 10^2^ PDAC#253, #354, PANC1 and L3.6pl cells in V-shaped ultra-low attachment 96-multiwell (Corning Costar), in complete PDAC growth medium (RPMI + 10% FBS) ± drugs (budesonide: from 0.001 to 20 µM; dexamethasone: from 0.001 to 20 µM; hydrocortisone: from 0.001 to 20 µM; 2-DG: from 0.5 to 50 mM; metformin: from 0.05 to 3 mM; rotenone: from 0.05 to 50 nM) or DMSO as a control, and allowed to aggregate for 120 h. Spheroid formation was monitored at 48, 72, 96 and 120 h after seeding, by measuring aggregates area, using ImageJ software. Spheroid volume was calculated by deriving the diameter from the area. Spheroid growth rate was calculated as the mean of the difference between the volume of a day and the volume of the previous day divided by the time.

$$\eqalign{ Spheroid\, & growth\,\,rate = \cr & {{\left({spheroid\,\,volum{e_{t2}}-spheroid\,\,volum{e_{t1}}} \right)} \over t} \cr}$$

### TMRE analysis

For evaluation of oxidative phosphorylation (OXPHOS), the TMRE Mitochondrial Membrane Potential Assay kit (Abcam) was used, according to manufacturer instructions. Briefly, PDAC spheroids ± budesonide were either directly stained with TMRE (25 nM), or dissociated using trypsin-EDTA for 30 min at 37 °C before staining. Both live spheroids and dissociated cells were stained with TMRE (25 nM) for 15 min and then analyzed by confocal microscopy (Nikon A1 microscope) and flow cytometry (FACS-ARIAIII, Becton-Dickinson), respectively. FCCP treatment was performed before staining with TMRE for 10 min at 37 °C. The NIS Element C (Nikon, Tokyo) software was used for image acquisition/elaboration.

### Cell cycle analysis of 3D tumor spheroids

To perform cell cycle analysis, 3-day old PDAC spheroids ± budesonide (1 µM) were dissociated with trypsin-EDTA for 30 min at 37 °C, before staining. Cells were then centrifuged for 5 min at 2000 rpm. Cell pellets were incubated in Sodium (Na)-Citrate buffer [0.1% Na-Citrate, 0.05% Nonidet P-40 (Sigma-Aldrich), 50 µg/ml Propidium Iodide and 0.2 µg/ml RNAse (Qiagen)] for 15 min, processed by flow cytometry, using a BD FACS-Canto™ II (Biosciences) and analyzed using the DivaTM software.

### Tumor spheroid inclusion and sectioning

Tumor spheroids were fixed for 48 h with 4% PFA. Post-fixed tumor spheroids were incubated in 30% sucrose (w/v) overnight in agitation, and embedded in OCT (Bio-Optica). Ten µm-sections were cut using a Leica Cryostat (Leica CM3050 S).

### Immunofluorescence analysis

Immunofluorescence was performed on PDAC cells obtained from either 2D cultures, cytospin samples or 3D spheroids. Cells from 2D cultures were fixed (4% PFA) and permeabilized (0.1% Triton X-100) for 10 min at RT and incubated with blocking solution (0.1% Triton X-100/5% BSA) for 1 h. Primary antibodies (listed in Table S1) were incubated overnight at 4 °C, followed by the respective secondary antibodies (Alexa Fluor Molecular Probes). For cytospin samples, dissociated cells were resuspended in 15% FBS/1X PBS and centrifuged at 800 rpm for 8 min using a Thermo Shandon Cytocentrifuge (CytoSpinTM 4), and fixed with 4% PFA for further analyses.

Tumor spheroids were fixed for 48 h with 4% PFA, permeabilized with PBSFT (10% FBS, 0.2% Triton X-100) for 30 min at 4 °C and incubated with PBSFT for 1 h at 4 °C in agitation. Primary antibodies (listed in Table S1) were incubated overnight at 4 °C with agitation followed by incubation with the appropriate secondary antibodies (Alexa Fluor Molecular Probes). Confocal images were acquired with a Nikon A1 microscope. The NIS Element C (Nikon, Tokyo) software was used for image acquisition/elaboration.

### Western blot

Total proteins were extracted in 20 mM Tris pH 8, 150 mM NaCl, 10 mM EDTA, 1% Triton X-100, 10% Glycerol, 1 mM Zinc acetate lysis buffer, resolved on SDS-PAGE gels and transferred onto PVDF membranes (iBlot dry Transfer System; Life Technologies). Primary Antibodies (listed in Table S1) were used overnight at 4 °C followed by the appropriate HRP-conjugated secondary antibodies. Detection was performed with ECL reagents (EuroClone). ImageJ software was used for the densitometric analysis.

### RNA extraction and qPCR

Total RNAs were extracted using the RNeasy kit or Trizol (Invitrogen) and reverse transcribed with the High Capacity cDNA Reverse Transcription Kit (Applied biosystems by Thermo Fisher Scientific). qPCR was performed using SYBR Green PCR master mix (FluoCycle IITM SYBR, EuroClone). Primers are listed in Table S2.

### RNA-Sequencing

For RNA Sequencing, PDAC#253 cells were treated ± budesonide in 2D and in 3D culture conditions (20 and 1 µM, respectively) for 3 days in PDAC growth medium (RPMI +10% FBS), as described above.

3’RNA-Seq (2D culture) and RNA-Seq (3D spheroids) were performed at Genomix4Life (https://www.genomix4life.com/en/bioinformatic_technologies.html) using Illumina platform. Raw data were aligned to HG38 - Release 37 (GRCh38.p13). Quantification of gene expression was performed using FeatureCounts (version 2.0). R software was used to create a matrix of all genes expressed in all samples with the corresponding read-counts, and the Bioconductor package DESeq2 was used to normalize the data, using the median of ratio, to perform the differential expression analysis (p value < 0.05). In particular, the counts were divided by sample-specific size factors determined by median ratio of gene counts relative to geometric mean per gene. Differential gene expression analysis was performed exclusively on protein-coding genes based on information in the gtf file available here https://ftp.ebi.ac.uk/pub/databases/gencode/Gencode_human/release_41/gencode.v41.annotation.gtf.gz. GSEA - Molecular Signature Database for Gene set enrichment - analysis was conducted using predefined genes signatures (Canonical pathways). Gene Ontology was performed using the David software (https://david.ncifcrf.gov/#).

The results of both the differential gene expression analysis and GSEA were integrated to gain further insights into underlying biological processes.

### Xenograft experiment

All studies were conducted in compliance with the Italian guidelines and approved by the local ethical review committee (IACUC #369/2021-PR). Animals (CD1 nude mice) were purchased from Charles River and maintained in pathogen-free animal facility and monitored daily at the IGB-CNR.

For subcutaneous tumor growth, 3 × 10^5^ PDAC#253 cells, resuspended in 100 µl 1XPBS were injected per flank of each mouse (*n*≥5 mice/per group). Three days after injection, each group of mice received vehicle DMSO or budesonide (3 mg/Kg) by intraperitoneal injection (i.p.). Seven days after injection, the third group of mice received gemcitabine (125 mg/Kg) i.p. Tumor growth was monitored every week by measuring tumor shortest (d) and longest (D) diameters with an electronic caliper. The volumes were calculated using the formula D × d^2^/2. For ethical reasons mice were sacrificed when tumors reach a maximum volume of 1,500–2,000 mm^3^.

### Immunohistochemistry

Formalin-fixed, paraffin-embedded (FFPE) mouse tumor tissue sections (10 μm) were incubated with 3% H_2_O_2_ for 5 min and then blocked with 10% goat serum and 1% BSA in PBS for 1 h. Samples were incubated with primary antibodies (listed in Table S1) overnight at RT in 10% goat serum and 1% BSA. HRP-conjugated secondary antibodies were used and signals were developed with DAB. Samples were counterstained with hematoxylin (Dako). Images were captured on a Nikon A1 microscope. The NIS Element C (Nikon, Tokyo) software was used for image acquisition/elaboration.

### Statistical analysis

The data were represented as mean ± standard deviation (SD) or ± standard error of the mean (SEM), as well as through boxplots or dotplots. The number of independent experiments is shown as “n” and the total sample size is provided in each figure legend. To assess differences, a two-tailed paired Student’s t-test was conducted, and significance was considered as *p* ≤ 0.05. Data representations, including graphs and boxplots, were generated using either Microsoft Excel or RStudio software, specifically version 1.1.463 from RStudio, Inc., accessible at https://www.rstudio.com/.

## Results

### Budesonide reduces the mesenchymal/invasive features of PDAC cells in 2D cultures without affecting cell proliferation

**Fig. 1:**
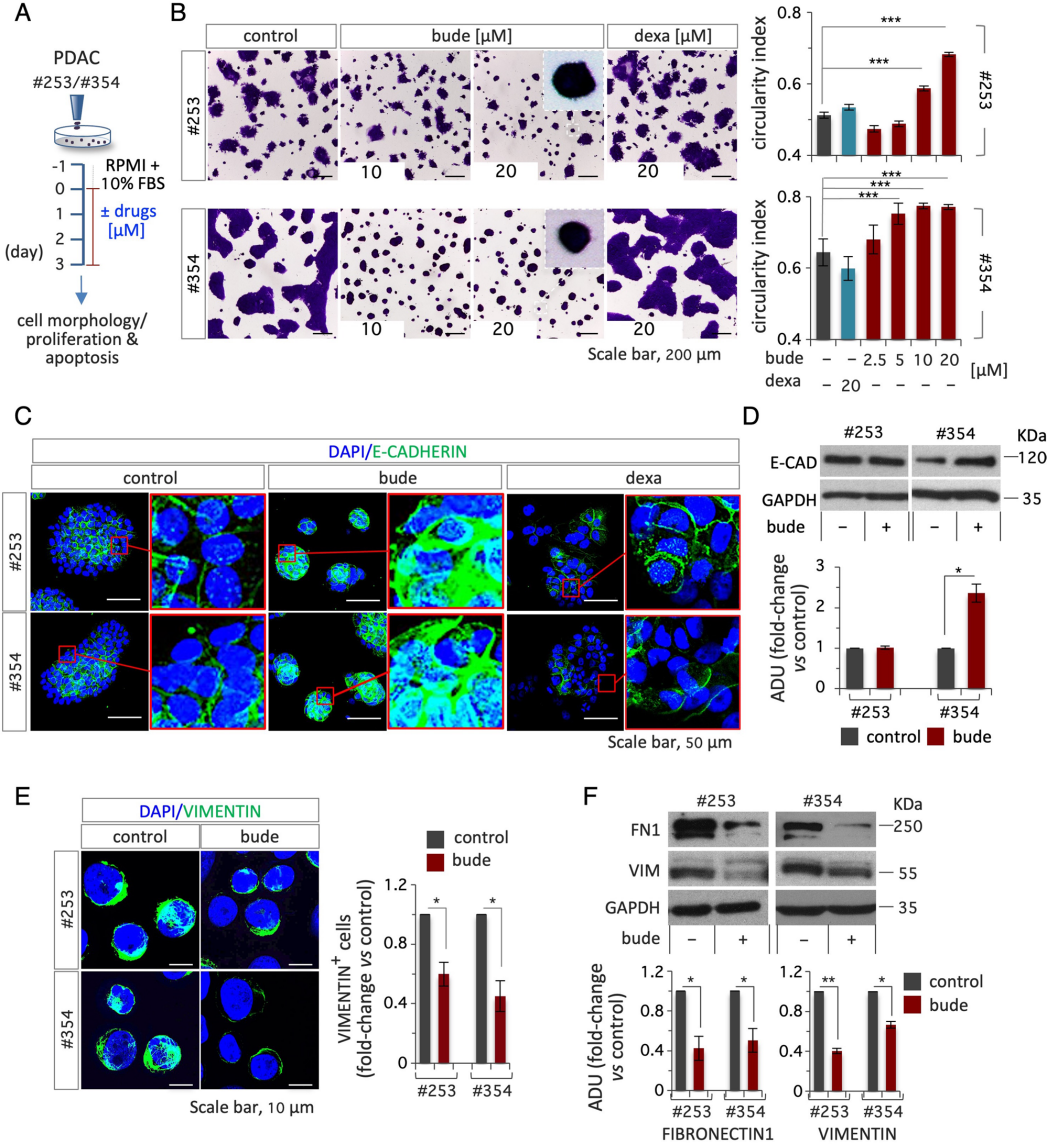
Budesonide promotes cellular adhesion in patient-derived PDAC cells. **A** Schematic representation of the experimental procedure. PDAC#253 and #354 cells were plated (1.5 × 10^4^ cells/cm^2^) on gelatin-coated plates at day − 1. On day 0, cells were treated ± budesonide (from 2.5 to 20 µM), dexamethasone (20 µM) or DMSO (control) for 3 days. **B** Representative pictures of PDAC cells treated with budesonide, dexamethasone or DMSO, at the indicated concentrations and stained with crystal violet (*left*) and quantification of the circularity index (*right*). **C** Representative confocal images of E-CADHERIN (green) staining in PDAC#253 and #354 cells treated ± budesonide (20 µM), or ± dexamethasone (20 µM). Nuclei were stained with DAPI (blue). **D** Western blot analysis (*upper*) and densitometric quantification (ADU; *bottom*) of E-CADHERIN in PDAC#253 and #354 treated from (**A)**. Densitometric analysis (ADU) is shown as fold-change vs. DMSO-treated cells, after normalization to GAPDH. **E** Representative confocal images of VIMENTIN (green) staining (*left*) and quantification of VIMENTIN^+^ cells (*right*) in PDAC#253 and #354 cells ± budesonide (20 µM). Nuclei were stained with DAPI (blue). **F** Representative western blot analysis (*upper*) and densitometric quantification (ADU; *bottom*) of FIBRONECTIN (FN1) and VIMENTIN in PDAC#253 and #354 cells treated ± budesonide (20 µM). ADU is shown as fold-change vs. DMSO-treated cells after normalization to GAPDH. Data are mean ± SEM (*p ≤ 0.05; **p ≤ 0.005; ***p ≤ 0.001; *n* = 3, Student’s t-test)

To investigate whether budesonide may directly affect PDAC growth and development, we first assessed its effect on tumor cell morphology and behavior using two independent patient-derived primary cell lines, PDAC#253 and #354 [15, 16]. To this end, PDAC cells were plated in 2D culture conditions in PDAC growth medium (RPMI +10% FBS) and treated with either budesonide, another glucocorticoid (dexamethasone), or DMSO as a vehicle control (Fig. 1A). After 3 days, the cells were stained with crystal violet and the colony morphology was analyzed. Control colonies showed the expected flat shaped morphology with cells spreading from the colony edges outwards. Colony morphology was largely modified by budesonide, showing a round-shaped/compacted phenotype and higher circularity index compared to controls (Fig. 1B). This phenotypic effect was observed at budesonide concentrations starting from 5 to 10 µM in PDAC#354 and #253 cells, respectively (Fig. 1B). Of note, dexamethasone did not affect PDAC colony morphology even at the highest concentration (20 µM) tested (Fig. 1B), thus suggesting a specific effect of budesonide. We thus assessed whether budesonide may affect cell proliferation and apoptosis in these culture conditions. To this end, PDAC cells ± budesonide were analyzed by flow cytometry using the 5-ethynyl-2-deoxyuridine (EdU) incorporation assay and Annexin V/Propidium iodide staining. The percentage of EdU positive as well as Annexin V/PI positive cells was comparable in control and budesonide-treated cells, suggesting that neither proliferation or apoptosis were affected in the presence of budesonide (Fig. S1A-D). These results were further confirmed with the commercially available pancreatic cancer cell line PANC1. Indeed, budesonide similarly modified the growth behavior of PANC1 cells (Fig. S1E-F), without affecting proliferation and apoptosis (Fig. S1G-H).

**Fig. 2:**
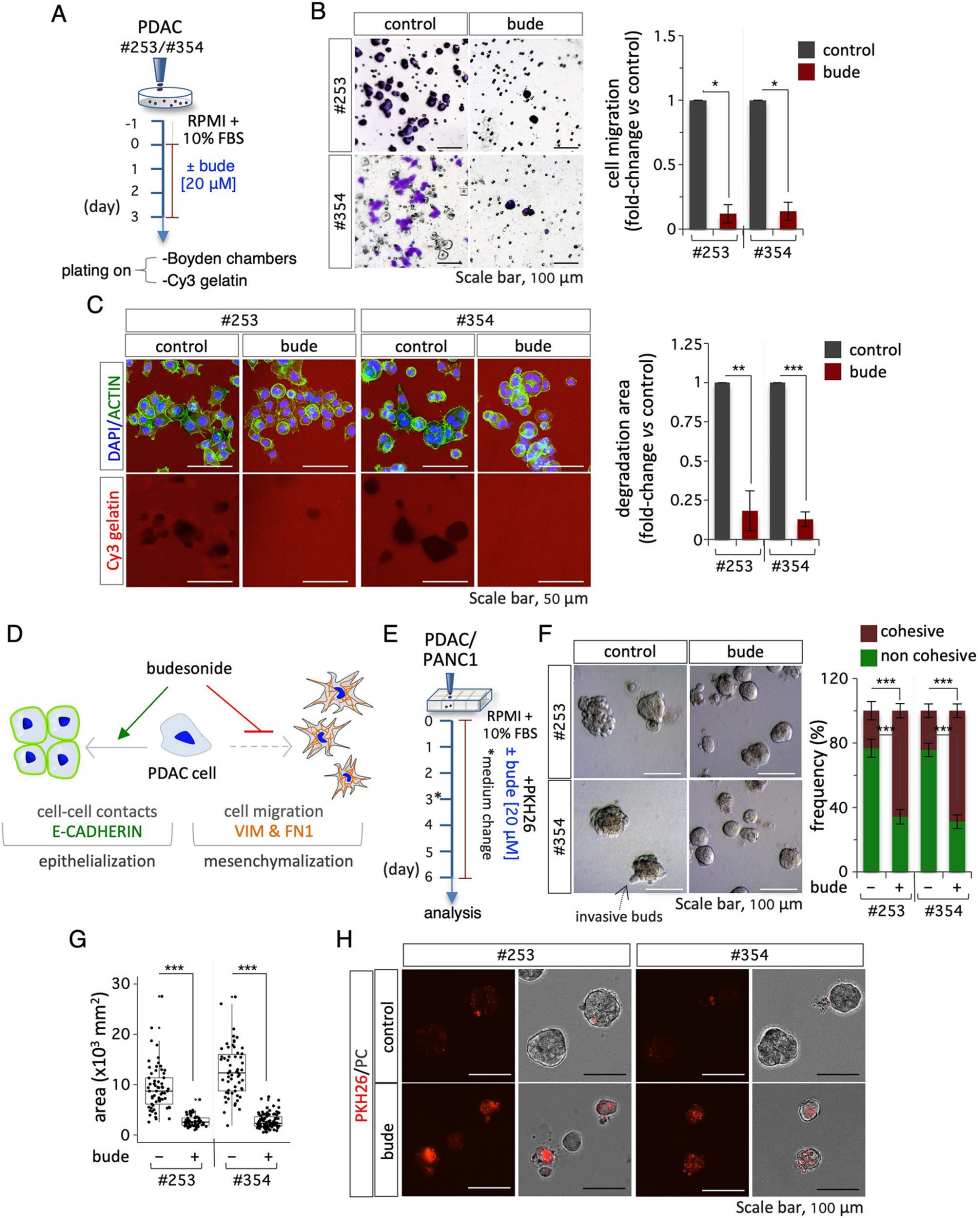
Budesonide reduces mesenchymal markers and PDAC cell migration and invasion. **A** Schematic representation of experimental procedure. PDAC#253 and #354 cells were plated (1.5 × 10^4^ cells/cm^2^) on gelatin-coated plates at day − 1. On day 0, cells were treated ± budesonide (20 µM) or DMSO (control). After 3 days, cells were dissociated and plated (2.5 × 10^4^ cells/well) on Boyden chambers or (1 × 10^5^ cells/cm^2^) on Cy3-conjugated gelatin. **B** Representative crystal violet images (*left*) and quantification (*right*) of PDAC#253 and #354 cells ± budesonide migrating through the transwell. Cell migration was quantified at 6 h (h) after seeding. Data are shown as fold-change vs. control (DMSO) and are mean ± SEM (*p < 0.05; *n* = 3, Student’s t-test). **C** Representative confocal images of ACTIN (green) staining (*left*) and quantification (*right*) of Cy3-gelatin degraded area in PDAC#253 and #354 cells ± budesonide. Invasion was quantified 6 h after seeding. Nuclei were counterstained with DAPI. Data are mean ± SEM (**p < 0.005, ***p < 0.001; *n* = 3, Student’s t-test) after normalization vs. the total number of nuclei. **D** Schematic representation of the effects of budesonide in 2D culture. Budesonide promotes epithelialization and reduces PDAC cell migration and invasion. **E** Schematic representation of 3D organotypic culture procedure. PDAC (#253 and #354) and PANC1 (1 × 10^3^ cells/cm^2^) cells were plated on a layer of 100% matrigel in complete medium containing 2% matrigel ± budesonide (20 µM) for 6 days. Medium was refreshed at day 3. **F-G** Representative phase-contrast images (**F**, *left*), frequency of cohesive vs. not cohesive structures (**F**, *right*) and quantification of the area (**G**) of PDAC (#253 and #354) spheroids ± budesonide at day 6 after plating. Data are mean ± SEM (**F**) or mean ± SD (**G**) (***p < 0.001; *n* = 4, Student’s t-test). **H** Representative images of PKH26-labeled (red) spheroids at day 6 post labeling derived from PDAC#253 and #354 cells treated ± budesonide (20 µM)

Recent findings showed that budesonide specifically promotes the stabilization of the cell-cell adhesive contacts in embryonic stem cells and triple negative breast cancer cells [11, 13]. To investigate this phenotype in PDAC cells, we analyzed the expression of epithelial and mesenchymal markers by immunofluorescence and western blot. We first assessed the effect of both budesonide and dexamethasone on the expression/localization of the adhesion protein E-CADHERIN in PDAC#354 and #253 cells. Immunofluorescence analysis showed increased accumulation of E-CADHERIN at the cell-cell junctions in budesonide- but not dexamethasone-treated PDAC cells compared to control, which correlates with changes in colony morphology (Fig. 1C). Of note, treatment with budesonide increased expression of E-CADHERIN protein in PDAC#354 but not #253 cells, likely reflecting their different genetic background (Fig. 1D). Conversely, budesonide reduced the expression of the mesenchymal markers VIMENTIN and FIBRONECTIN (FN1) both in PDAC and PANC1 cells (Fig. 1E-F, and Fig. S1I), providing molecular support to the hypothesis that budesonide induces epithelial features in pancreatic cancer cells. To further investigate this phenotype, we analyzed cell migration and invasion by transwell assay and fluorescent gelatin degradation assay in vitro (Fig. 2A). Budesonide significantly reduced PDAC#253 and #354 cell migration (∼10 times) in response to serum gradients in the boyden chamber assay (Fig. 2B). Furthermore, budesonide reduced by more than 80% the capacity of both PDAC and PANC1 cells to degrade and invade Cy3-fluorescent gelatin (Fig. 2C, Fig. S2B-C). In contrast, dexamethasone did not affect the invasive capacity of PDAC and PANC1 cells, further supporting the idea that budesonide exerts a specific activity (Fig. S2A, Fig. S2B-C).

Altogether our findings indicate that budesonide promotes an epithelial phenotype and antagonizes the mesenchymal state in PDAC cells in 2D settings and suggest that it inhibits their ability to migrate and invade the extracellular matrix, without affecting proliferation (Fig. 2D).

### Budesonide inhibits PDAC cell proliferation in a three-dimensional environment in vitro and in vivo

While 2D cultures have been largely used to study cancer cell biology, they do not reproduce the three-dimensional cell-cell/cell-matrix interactions proper of the tumor microenvironment [19]. This is particularly relevant when evaluating the effect of a drug on tumor growth. Thus, to investigate the effect of budesonide in a more physiological context, we used 3D organotypic cultures of pancreatic cancer cells. To this end, PDAC cells were seeded as single cells onto a layer of 100% matrigel and incubated in PDAC growth medium (RPMI +10% FBS) with 2% matrigel, either alone or in the presence of budesonide (20 µM), and cultured for 6 days (Fig. 2E). In the presence of budesonide PDAC spheroids were more homogeneous and compacted compared to control (DMSO) (Fig. 2F). Specifically, budesonide increased the fraction (up to 70%) of cohesive spheroids compared to DMSO, which conversely showed a higher fraction of irregular/non-cohesive spheroids with invasive buds (Fig. 2F and Fig. S2D). Comparable results were obtained with 3D organotypic cultures of PANC1 cells (Fig. S2E). Of note, quantification of the spheroid area showed that it was significantly reduced in the presence of budesonide compared to controls (Fig. 2G), raising the hypothesis that budesonide could exert an anti-proliferative effect in 3D culture conditions. To further address this issue, we stained the spheroids with the membrane dye PKH26, which dilutes as the cells divide (Fig. 2E). Quite unexpectedly, while control spheroids lost PKH26 staining at day 6, budesonide-treated spheroids retained the membrane dye (Fig. 2H), indicating that proliferation is significantly reduced in this condition.

**Fig. 3:**
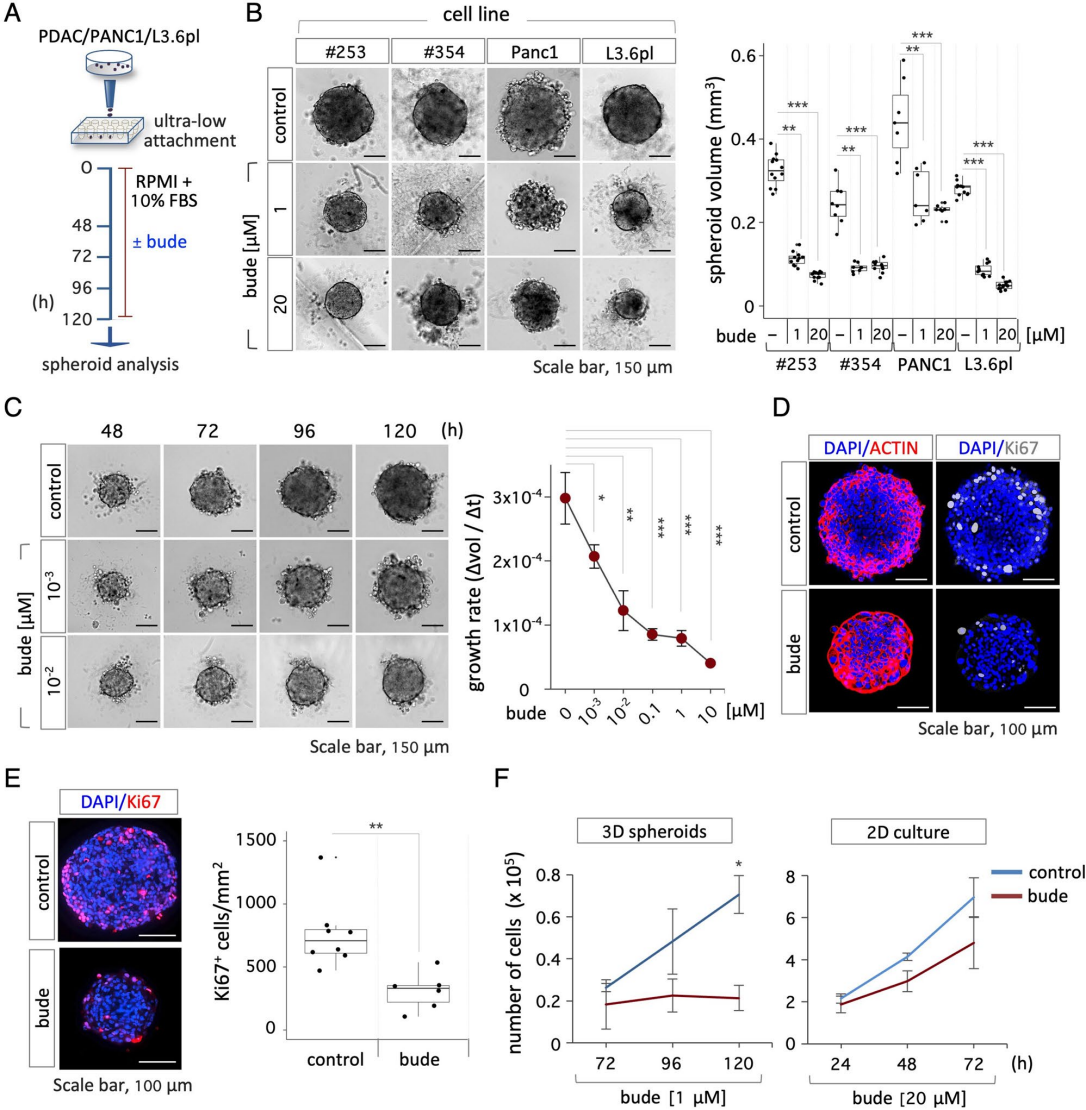
Budesonide reduces the growth of pancreatic cancer cell spheroids. **A** Schematic representation of the experimental design. PDAC (#253 and #354), PANC1 and L3.6pl cells were seeded (5 × 10^2^ cells/well) in ultra-low attachment plates ± budesonide (from 10^− 3^ µM to 20 µM) or DMSO (control) for 5 days. **B** Representative pictures (*left*) of PDAC (#253, #354), PANC1 and L3.6pl spheroids ± budesonide (1 and 20 µM) and quantification (*right*) of the spheroid volume. Data are mean ± SD (**p < 0.005, ***p < 0.001; *n* = 3, Student’s t-test). **C** Representative pictures (*left*) and growth rate (*right*) of PDAC#253 spheroids ± budesonide calculated as the mean of the ratio between the Δvolume and the Δtime (48, 72, 96 and 120 h). Data are mean ± SEM (*p < 0.05; **p < 0.005, ***p < 0.001; *n* = 3, Student’s t-test). **D** Representative confocal images of ACTIN (red) and Ki67 (grey) staining in PDAC#253 spheroids ± budesonide (1 µM). Nuclei were counterstained with DAPI (blue). **E** Representative images (*left*) and quantification (*right*) of Ki67 (red) staining in cryo-sections of PDAC#253 spheroids. Nuclei were counterstained with DAPI (blue). The number of Ki67^+^ cells/area is shown as mean ± SD (**p < 0.005; *n* = 3, Student’s t-test). **F** Time course quantification of cell number in 3D spheroids (*left*) and 2D cultures (*right*) of PDAC#253 cells treated ± budesonide at the indicated concentrations. Data are mean ± SD (*p < 0.05; *n* = 3, Student’s t-test)

To better investigate the effect of budesonide on pancreatic cancer spheroid growth and improve reproducibility, we set up a robust spheroid formation assay by seeding pancreatic cancer cell lines (500 cells/well) into V-shaped ultra-low attachment 96-multiwell plates in PDAC growth medium (RPMI + 10 % FBS), previously used in both 2D and organotypic cultures, without supplementation of growth factors and cytokines. Our initial efforts to generate pancreatic cancer spheroids were unsuccessful due to a reduced propensity of these cells to aggregate, which has already been described [20]. Thus, in the attempt to favor the cell-cell vs. cell-substrate adhesive contacts, cells were passaged at a higher ratio (1:2 vs. 1:6) before seeding (500 cells/well) into V-shaped ultra-low attachment plates (Fig. 3A). This adaptive approach improved the ability of different pancreatic cancer cell lines, including PDAC, PANC1 and the highly aggressive and metastatic L3.6pl cells, to generate floating, round-shaped, compacted cell aggregates that were homogeneous in size, ranging around a mean value of 0.032 ± 0.004 mm^3^ (Fig. 3B). To assess the effect of budesonide on PDAC, PANC1 and L3.6pl spheroids, cells were allowed to aggregate with either budesonide at 1 or 20 µM or DMSO as a control. Quantification of the spheroid volume revealed that budesonide-treated spheroids were significantly smaller in size compared to controls (Fig. 3B). We thus performed a dose-dependent assay to evaluate the effect of lower concentrations of budesonide (from 10 to 10^− 3^ µM) on spheroid growth, calculating the change in volume over time (Δvol/Δt). Results showed a clear dose-dependent response to budesonide, which significantly reduced the growth rate of PDAC spheroids (∼3 times) compared to controls, up to a concentration of 10^− 2^ µM (Fig. 3C, Fig. S3A). Double immunofluorescence staining for the nuclear protein Ki67 and the cytoskeleton protein ACTIN on both whole mount spheroids (Fig. 3D, Fig. S3B) and spheroid cryo-sections (Fig. 3E), showed a significant reduction of Ki67^+^ cells in budesonide-treated spheroids, which were smaller and highly compacted compared to controls. Complementary to these findings, we showed that both the diameter of the spheroids and the number of cells/spheroid diameter were significantly reduced in the presence of budesonide compared to controls (Fig. S3C). To further investigate this phenotype, we compared the growth rate of PDAC cells ± budesonide in 2D and 3D cultures. PDAC cells seeded in 2D culture plates and treated with budesonide at the highest concentration (20 µM) showed a similar growth rate compared to controls (Fig. 3F, Fig. S3D). Conversely, the doubling time of PDAC spheroids strongly increased in the presence of budesonide (1 µM) from ∼40 h to > 120 h (Fig. 3F, Fig. S3D), further supporting the hypothesis that budesonide exerts an anti-proliferative effect on PDAC cells cultured in 3D conditions.

**Fig. 4:**
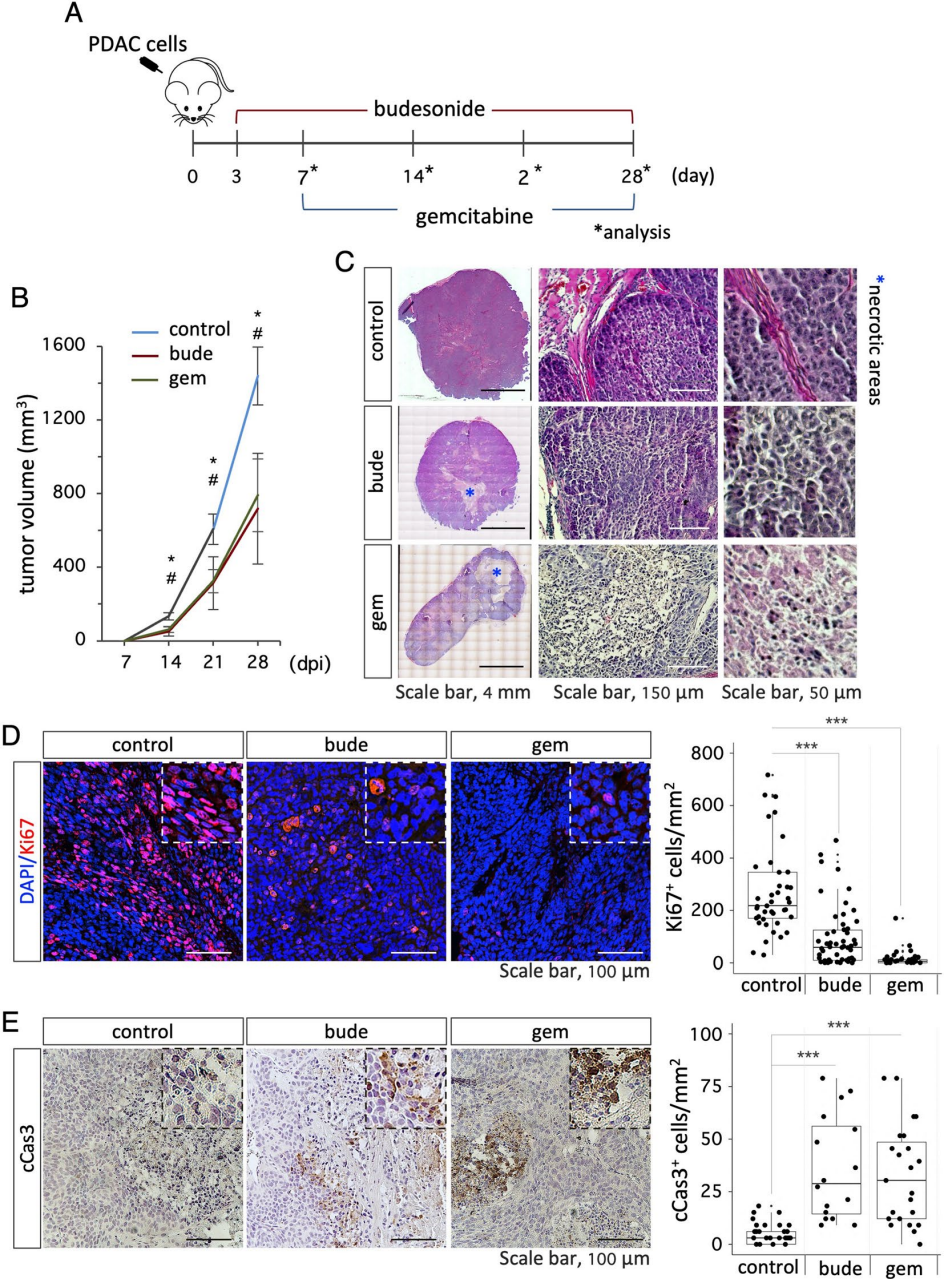
Budesonide reduces PDAC tumor growth in vivo. **A** Schematic representation of the experimental procedure. CD1 athymic nude mice were subcutaneously injected with PDAC#253 cells (3 × 10^5^ cells/flank) and injected intraperitoneally (i.p.) with either budesonide (3 mg/Kg) everyday, vehicle (control) or gemcitabine (125 mg/Kg) 2 times/week. **B** Quantification of tumor volume (mm^3^) in mice injected with budesonide (bude), gemcitabine (gem) or vehicle (control). Data are mean ± SEM. *n*≥ 5 mice/group; *n*≥ 10 tumors/group. # and * indicate the significance (*p* < 0.05) of budesonide (bude) and gemcitabine (gem) vs. control (vehicle), respectively. dpi = days post inoculations. **C** Representative images of H&E-stained tumor sections from control (vehicle), budesonide (bude)- and gemcitabine (gem)- treated mice. Mosaic reconstruction (*left*) and higher magnifications (*middle* and *right*) of tumor sections are shown. Asterisks indicate necrotic areas. **D** Representative images (*left*) of Ki67 staining (red) and quantification (*right*) of Ki67^+^ cells in tumor sections from budesonide (bude)-, gemcitabine (gem)- and vehicle (control)- treated mice. Nuclei were counterstained with DAPI (blue). **E** Representative images (*left*) of cleaved Caspase3 (cCas3) immunohistochemistry and quantification (*right*) of cCas3^+^ cells in tumor sections from budesonide (bude)-, gemcitabine (gem)- and vehicle (control)-treated mice. Data are mean ± SD (***p < 0.001; *n* = 10–12 tumors/group)

These unexpected findings prompted us to investigate the effect of budesonide in the tumor microenvironment in vivo. To this end, we established a human PDAC xenograft model by injecting PDAC cells subcutaneously into the flanks of CD1 nude mice. Mice were divided into three groups and were injected intraperitoneally (i.p.) with DMSO-vehicle as a control, budesonide (3 mg/Kg; 6 days/week) or gemcitabine (125 mg/Kg; twice/week), which is the gold-standard treatment for PDAC (Fig. 4A). Tumor volume was measured every week and mice were sacrificed at week 4. Budesonide- and gemcitabine- treated mice formed tumors of significantly smaller volumes compared to control mice (control 1439 ± 157 vs. budesonide 718 ± 301 vs. gemcitabine 791 ± 198 mm^3^; Fig. 4B, Fig. S4A). However, tumors from the 3 groups were similar in their composition, i.e., solid tumor of high grade/poor differentiation, with variable degree of necrosis, ranging from 0 to 40% (Fig. 4C). Immunofluorescence analysis of tumor sections revealed that budesonide strongly reduced the number of proliferating Ki67^+^ cells compared to control, similarly to gemcitabine (Fig. 4D, Fig. S4B). Furthermore, staining with cleaved CASPASE3 (cCAS3) showed a significant increase of cCAS3^+^ cells both in budesonide and gemcitabine groups compared to control, suggesting increased apoptosis upon treatment with both budesonide or gemcitabine (Fig. 4E, Fig. S4C).

All together our findings reveal that budesonide exerts a previously unidentified anti-proliferative effect on PDAC cells, exclusively under 3D growth conditions.

### The anti-proliferative effect of budesonide on PDAC spheroids requires an active GC-GR axis

**Fig. 5:**
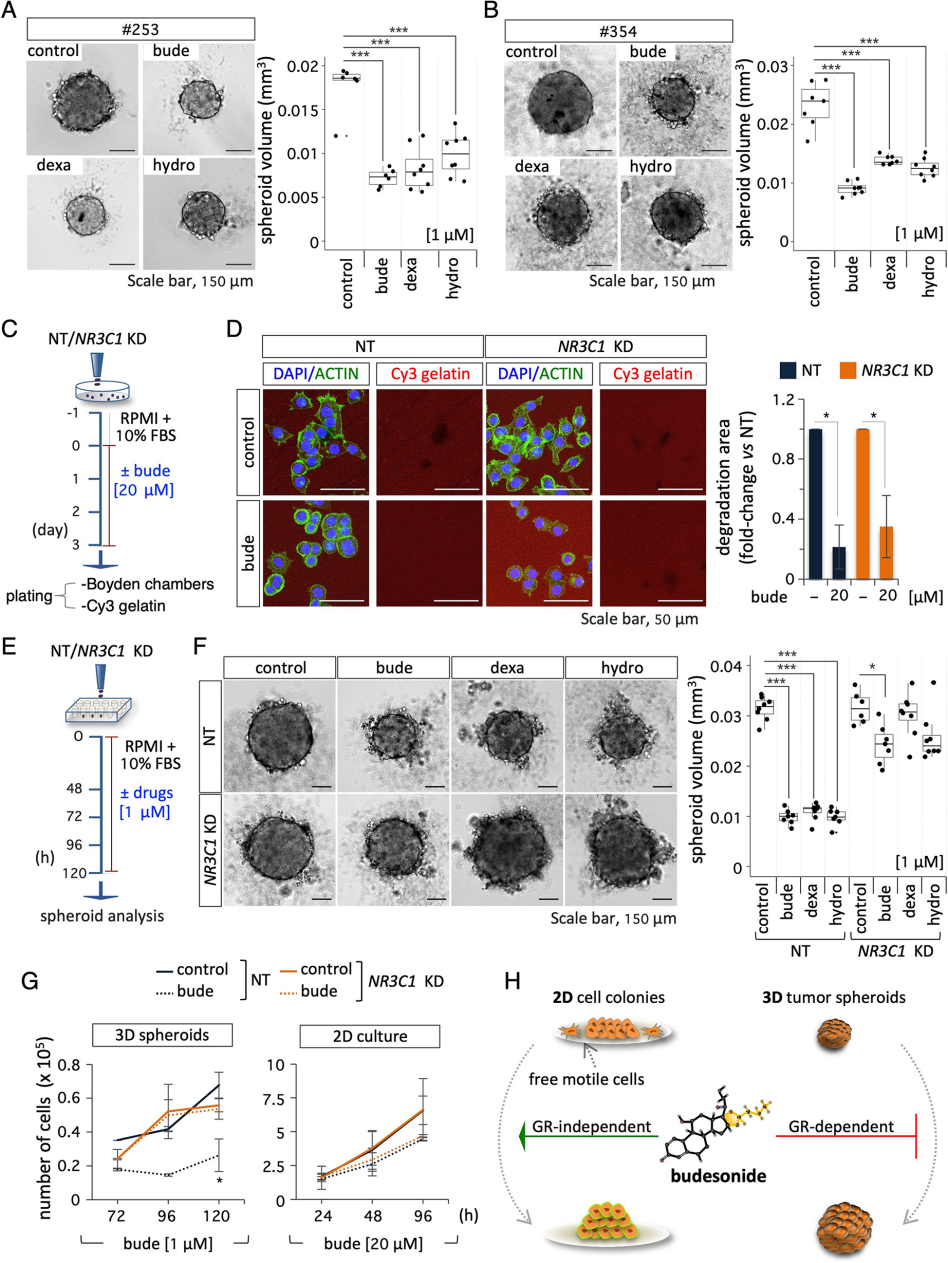
Budesonide-dependent reduction of PDAC spheroid volume is Glucocorticoid Receptor (GR)-dependent. **A-B** Representative pictures (*left*) and volume quantification (*right*) of spheroids from PDAC#253 **A** and #354 **B** cells ± budesonide (bude), dexamethasone (dexa) or hydrocortisone (hydro) at 1 µM. DMSO was used as a control. Data are mean ± SD (***p < 0.001; *n* = 3, Student’s t-test). **C** Schematic representation of experimental procedure. NT (control/ShEmpty) and *NR3C1* KD PDAC#253 cells were plated (1.5 × 10^4^ cells/cm^2^) on gelatin-coated plates at day − 1. On day 0, cells were treated ± budesonide (20 µM). After 3 days in culture, cells were dissociated and plated (1 × 10^5^ cells/cm^2^) on Cy3-gelatin. **D** Representative confocal images (*left*) of ACTIN staining (green) in NT and *NR3C1* KD PDAC#253 cells ± budesonide (20 µM) and quantification (*right*) of Cy3-gelatin degraded area. Nuclei were counterstained with DAPI. Data are mean ± SEM (*p < 0.05, *n* = 3, Student’s t-test), after normalization vs. the total number of nuclei. **E** Schematic representation of the experimental design. Control (NT) and *NR3C1* KD PDAC#253 cells were seeded in ultra-low attachment plates (5 × 10^2^ cells/well) and treated ± budesonide, dexamethasone, hydrocortisone (1 µM) or DMSO for 5 days. **F** Representative pictures (*left*) and volume quantification (*right*) of spheroids generated from control and *NR3C1* KD PDAC#253 cells treated with budesonide (bude), dexamethasone (dexa), hydrocortisone (hydro) at 1 µM or DMSO as control. Data are mean ± SD (*p < 0.05; ***p < 0.001; *n* = 3, Student’s t-test). **G** Time course analysis of NT and *NR3C1* KD PDAC#253 cell proliferation in 3D spheroids ± budesonide (*left*; bude: 1 µM), and in 2D cultures ± budesonide (*right*; bude: 20 µM). Data are mean ± SD (*p < 0.05; *n* = 3, Student’s t-test). **H** Schematic representation of the GR-independent and -dependent effects of budesonide. In 2D cultures, budesonide (> 2.5 µM) promotes epithelialization and reduces PDAC cell migration independently from the GR. In PDAC spheroids (3D), nanomolar concentrations of budesonide (≤ 10^− 2^µM) exert a GR-dependent anti-proliferative effect

It is well known that glucocorticoids (GCs), like budesonide, may exert both genomic and non-genomic effects, which are either dependent on or independent of the glucocorticoid receptor (GR), respectively. To investigate the underlying mechanism of budesonide activity on PDAC spheroids, we first assessed the effect of other GCs, such as dexamethasone and hydrocortisone. To this end, PDAC spheroids were generated in the presence of either budesonide, dexamethasone, hydrocortisone, or DMSO as a vehicle control. Quantification of the spheroid volume showed that both dexamethasone and hydrocortisone (used at 1 µM) significantly reduced the volume of PDAC#253 and #354 spheroids (Fig. 5A-B). Of note we found a clear dose-dependent response even at lower concentrations of GCs, up to 10^− 3^ µM in both cell lines (Fig. S5A-B). To directly assess the role of the GR, we silenced its expression in PDAC#253 cells by using an shRNA that targets the *NR3C1* gene encoding the GR [18]. We first verified efficient downregulation of *NR3C1* at both the RNA and protein level (Fig. S5C), and then tested the effect of budesonide on *NR3C1* KD (sh*NR3C1)* and NT (control/ shEmpty) cells in the different culture conditions. In 2D culture, budesonide significantly increased the circularity index of both control and *NR3C1* KD PDAC cell colonies (Fig. S5D). Furthermore, budesonide reduced the migration (Fig. 5C, Fig. S5E) and invasion (Fig. 5C-D) of *NR3C1* KD PDAC cells as efficiently as control cells, suggesting that the anti-migratory and invasive activities of budesonide are GR-independent and could be ascribed to non-genomic mechanisms.

We then assessed the effect of *NR3C1* KD on PDAC tumor spheroids (Fig. 5E). As expected, control (NT) PDAC spheroid volume was significantly reduced in the presence of budesonide as well as in the presence of dexamethasone and hydrocortisone; however, this effect was blunted in *NR3C1* KD tumor spheroids (Fig. 5F), suggesting that the anti-proliferative effect of budesonide in 3D spheroids relies on the GR. This hypothesis was further supported by the observation that budesonide reduced the doubling time of control but not of *NR3C1* KD PDAC spheroids (Fig. 5G). Conversely, budesonide did not affect the doubling time of both control and *NR3C1* KD cells in 2D culture conditions (Fig. 5G) even though the GR is expressed in this condition (Fig. S5C).

All together these data indicate that the anti-proliferative effect of budesonide on PDAC spheroids is GR-dependent, and that it is shared by other GCs (Fig. 5H).

### Genome-wide transcriptome profiling reveals 3D-induced metabolic remodeling of PDAC cells

**Fig. 6:**
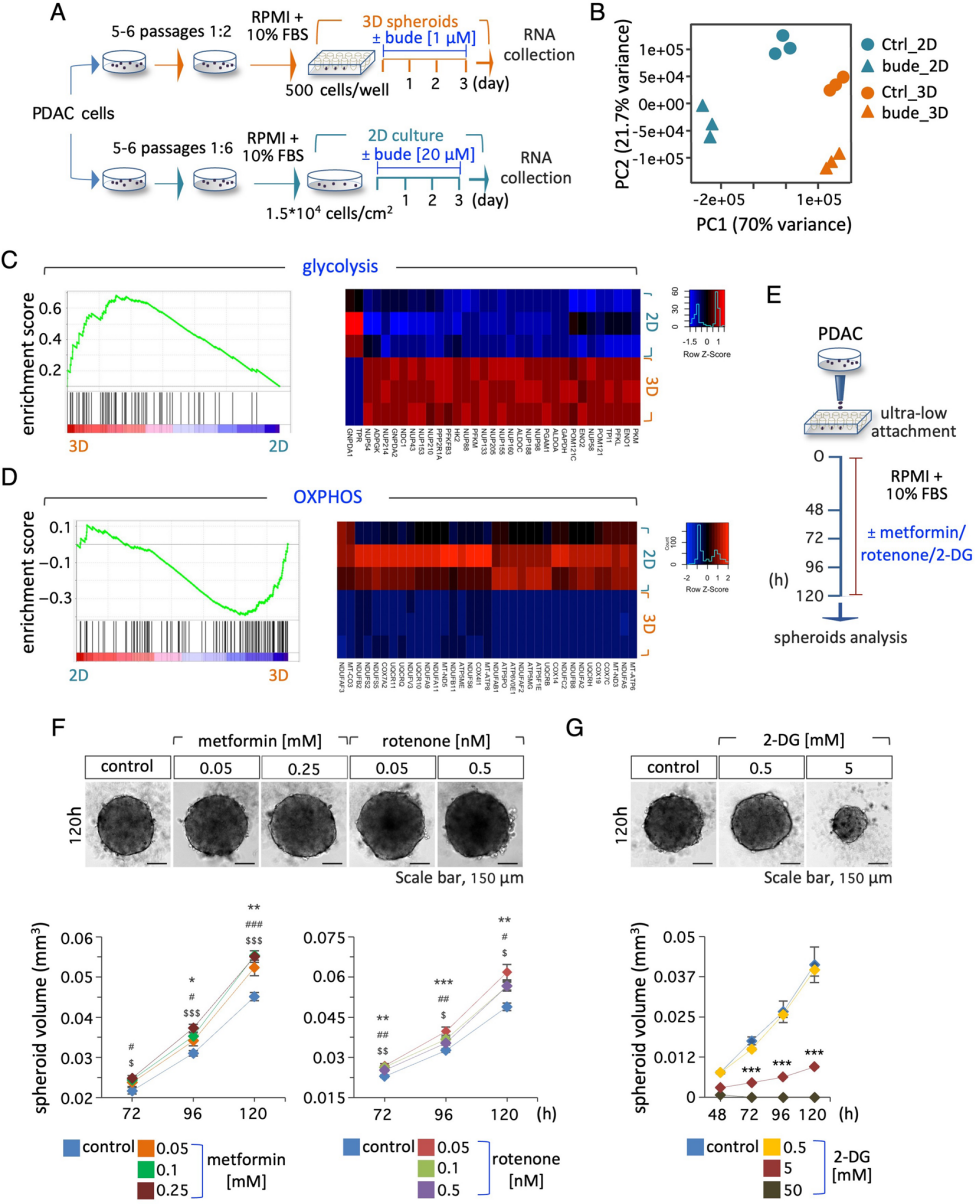
Transcriptome profiling of PDAC cells in 2D culture and 3D spheroids and sensitivity to energy metabolism inhibitors. **A** Schematic representation of the experimental procedure. PDAC#253 were either seeded in ultra-low attachment plates to form 3D spheroids or plated in 2D and treated ± budesonide at the indicated concentrations. DMSO was used as control. After 3 days, spheroids/cells were collected and RNA was extracted for RNA-Seq analysis. **B** Principal component analysis (PCA) of PDAC ± budesonide in 2D and in 3D culture. **C, D** GSEA plots related to glycolysis (**C**, *left*) and OXPHOS (**D**, *left*) positively and negatively enriched in 3D spheroids, respectively. Heatmaps of DEGs related to glycolysis (**C**, *right*) and OXPHOS (**D**, *right*) in control PDAC cells in 2D vs. 3D culture. **E** Schematic representation of the experimental design. PDAC cells were seeded (5 × 10^2^ cells/well) in ultra-low attachment plates ± metformin, rotenone, 2-DG or DMSO (control) for 5 days. **F** Representative pictures (120 h) of PDAC spheroids (*upper*) ± metformin (0.05 and 0.25 mM) or rotenone (0.05 and 0.5 nM) at the indicated concentrations, and time course analysis (*bottom*) of spheroid volume at the indicated time points. DMSO was used as control. Data are mean ± SEM (*, ^#^ and $ indicate the significance of metformin- or rotenone- treated cells vs. control *p < 0.05, **p < 0.005, ***p < 0.001; *n* = 3, Student’s t-test). **G** Representative pictures (120 h) of PDAC spheroids (*upper*) ± 2-DG (0.5 and 5 mM), and time course analysis (*bottom*) of spheroid volume at the indicated time points. DMSO was used as control. Data are mean ± SEM (***p < 0.001; *n* = 3, Student’s t-test)

To gain insight into the mechanism by which budesonide affects PDAC cell behavior in 2D and 3D cultures, we performed RNA-Seq analysis of PDAC cells grown on gelatin-coated plates (2D) and as spheroids (3D) ± budesonide (Fig. 6A).

Principal component analysis (PCA) of RNA-Seq data showed that the cells in the different conditions clustered apart (Fig. 6B). Comparison of the transcriptome profiles of control PDAC cells in 2D and 3D conditions revealed ∼6200 deregulated protein-coding genes (DE; fold change ≥ 1.5; padj ≤ 0.05; Fig. S6A). Gene ontology (GO) analysis (David software; https://david.ncifcrf.gov/#) showed that the up-regulated genes in tumor spheroids were enriched in key biological processes such as cell cycle, cytoskeleton and chromatin organization (Fig. S6B), whereas the down-regulated genes were primarily enriched in metabolic and biosynthetic processes, and OXPHOS (Fig. S6B). Gene set enrichment analysis (GSEA) confirmed a significant enrichment in genes involved in energy metabolism (Fig. 6C-D, Table S3). Among the key upregulated genes in 3D cultures that contributed to the GSEA results were glycolytic enzymes genes, including *HK2*, *PFKL*, *ALDOA*, *GAPDH*, *PGAM1*, *ENO1*, *PKM* (Fig. 6C). Conversely, several genes of the *NDUF*, *COX*, *ATP* and *UQCR* gene families that encode enzymatic complexes involved in the OXPHOS/electron transport were down-regulated in 3D growing cells (Fig. 6D, Table S4).

Interestingly, different gene sets involved in lipid metabolism (e.g., Sterol regulatory proteins SREBP, glycerophospholipid and cholesterol metabolism) (Fig. S6C, Table S3) were enriched in 3D tumor spheroids, while gene sets related to protein metabolism (e.g., response to amino acid starvation, ribosome, ribosomal proteins, translation elongation and initiation) were conversely depleted (Fig. S6C, Table S3). Altogether these results suggest that PDAC cells undergo a general metabolic remodeling to meet their needs to grow in a 3D environment. To investigate this hypothesis, we focused on energy metabolism, and evaluated the sensitivity of PDAC cells to inhibition of OXPHOS and glycolysis. Surprisingly, the growth of PDAC spheroids increased in the presence of sublethal concentrations of the OXPHOS inhibitors metformin and rotenone compared to controls (Fig. 6E-F, Fig. S6D). However, as expected, higher concentrations of these inhibitors reduced PDAC spheroid growth and showed a toxic effect (Fig. S6E, F). Conversely, PDAC spheroids were highly sensitive to sublethal concentrations (5 mM) of the glycolysis inhibitor 2-deoxyglucose (2-DG) (Fig. 6E and G, Fig. S6G).

Together, these data support the transcriptome results and suggest that PDAC spheroids mostly rely on glycolysis to sustain their growth.

### Budesonide modifies the metabolism of PDAC cells

**Fig. 7:**
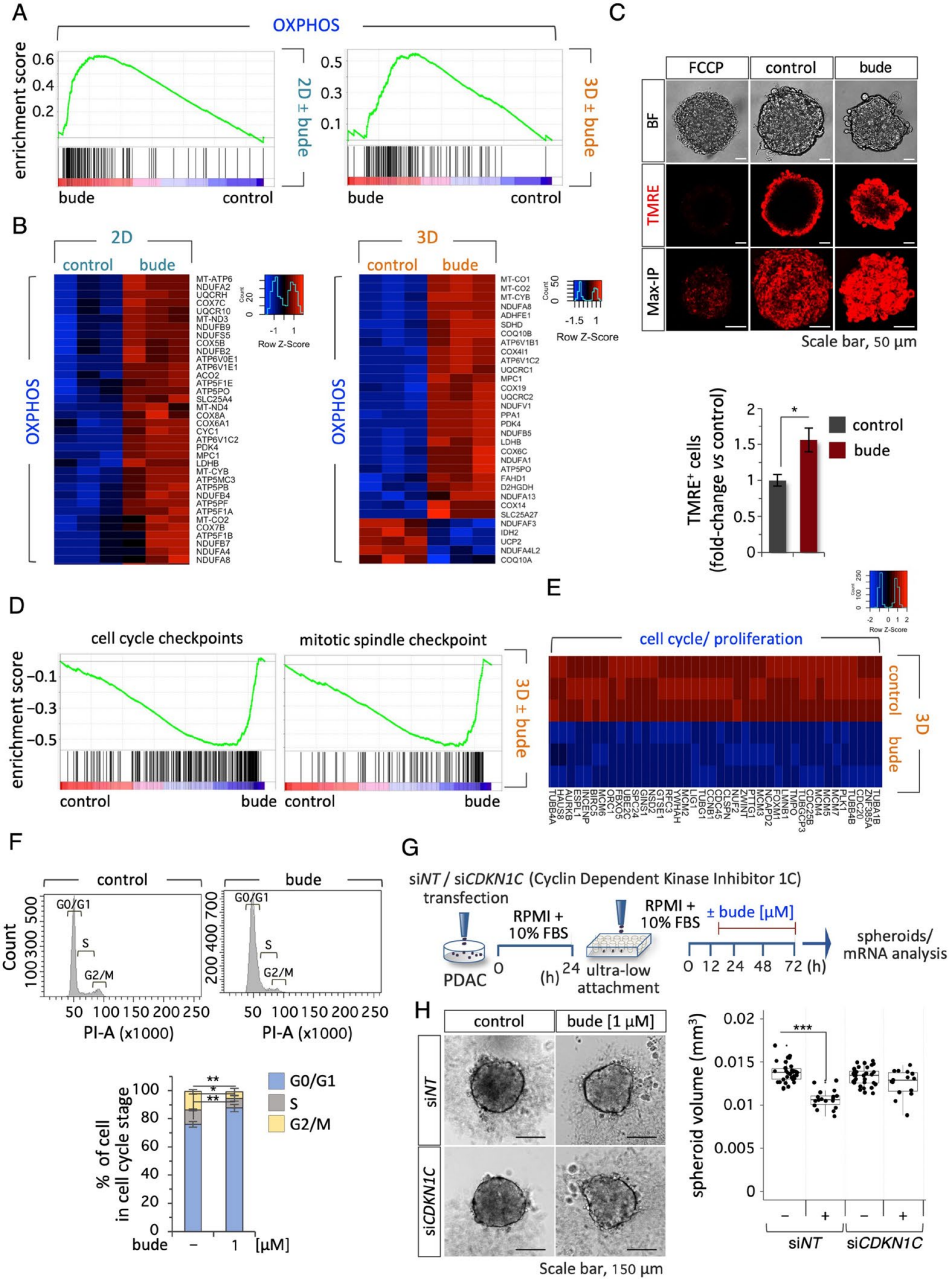
Budesonide induces metabolic remodeling in PDAC cells. **A** GSEA plots related to OXPHOS of positively enriched gene sets in budesonide (bude)- treated PDAC#253 in 2D (*left*) and 3D (*right*) culture. **B** Heatmaps of DEGs related to OXPHOS between control (DMSO) and budesonide-treated (bude) PDAC cells in 2D (*left*) and 3D (*right*) cultures. **C** Representative confocal images (*upper*) of TMRE staining (red) in PDAC spheroids ± budesonide (1 µM). Bright field (BF), TMRE and 3D Maximum projection intensity (Max-IP) are shown. Quantification by FACS analysis (*bottom*) of the percentage of TMRE^+^ PDAC cells ± budesonide (1 µM) in 3D culture. Data are shown as fold-change vs. control (DMSO) and are mean ± SEM (*p < 0.05; *n* = 3, Student’s t-test). **D** GSEA plots related to cell cycle checkpoint (*left*) and mitotic spindle checkpoint (*right*) of negatively enriched gene sets in budesonide-treated (bude) PDAC cells in 3D culture. **E** Heatmap of DEGs related to cell cycle and proliferation between control and budesonide-treated PDAC cells in 3D culture. **F** Representative flow cytometric histogram plots (*upper*) and quantification (*bottom*) of cell cycle analysis of PDAC spheroids ± budesonide (1 µM). Data are mean ± SEM (*p < 0.05, **p < 0.005; *n* = 3, Student’s t-test). **G** Schematic representation of the experimental procedure. PDAC#253 cells were transfected with a siRNA targeting *CDKN1C* or with a non-targeting siRNA (siNT) as control. After 24 h, cells were seeded (500 cells/well) in ultra-low attachment 96-well plates, and treated ± budesonide (1 µM). DMSO was used as control. **H** Representative pictures (*left*) and quantification (*right*) of spheroid volume of si*CDKN1C* and siNT PDAC spheroids ± budesonide (1 µM). Data are mean ± SD (***p < 0.001; *n* = 3, Student’s t-test)

GCs are well-known regulators of cell metabolism [21]. We thus hypothesized that budesonide could interfere with the observed metabolic remodeling induced in PDAC cells cultured in 3D. To gain insight into the inhibitory effects of budesonide on PDAC spheroids, we compared the transcriptome profiles of PDAC cells (2D) and spheroids (3D) ± budesonide (Fig. 6A-B). Differential expression (DE) analysis revealed 1591 and 3304 DE protein-coding genes (fold change ≥ 1.5; padj ≤ 0.05) in budesonide-treated 2D cells and tumor spheroids (3D), respectively (Fig. S6A). Of note, GO analysis of the down-regulated genes revealed a significant enrichment in terms related to cell migration, locomotion, EMT and ECM organization only in 2D culture conditions (Fig. S7A). Furthermore, GSEA analysis showed that genes involved in ECM organization, including ECM glycoproteins, were negatively enriched in budesonide-treated PDAC cells (Fig. S7B and Table S3). These data provide molecular support to the results that budesonide inhibits PDAC ability to migrate and invade the extracellular matrix in 2D culture (Fig. 2). Interestingly, GSEA analysis of all differentially expressed (DE) genes in budesonide-treated cells revealed a significant enrichment in genes involved in energy metabolism (OXPHOS and respiratory electron transport chain), in both 2D and 3D conditions (Fig. 7A-B, Fig. S7A-B and Table S3-S4) and depletion of those related to protein metabolism (response to starvation, translation initiation and elongation, and ribosome) (Table S3).

These results showing that budesonide induced the expression of genes involved in OXPHOS in 2D and 3D cultures (Fig. 7A-B), and our findings that PDAC cells undergo a metabolic reprogramming towards glycolysis in 3D spheroids (Fig. 6C), led us to hypothesize that budesonide may interfere with the metabolic remodeling that is specifically required for PDAC cells to grow in 3D. This may explain, at least in part, the fact that budesonide inhibits PDAC cell proliferation, exclusively within a 3D environment. To directly investigate this hypothesis, we evaluated the mitochondrial activity of PDAC spheroids ± budesonide by using tetramethylrhodamine, ethyl ester (TMRE), a positively-charged dye that marks mitochondria with membrane potential. Both confocal images and quantification by FACS analysis showed that budesonide significantly increased the fraction of TMRE^+^ cells compared to controls (Fig. 7C, Fig. S7C), which is in line with the idea that GCs are inducers of mitochondrial activity and gluconeogenesis [21]. Furthermore, the expression of genes involved in OXPHOS metabolism like *PDK4* and *ATP6V1C2* was strongly induced by budesonide in control (NT/ shEmpty) but not in *NR3C1* KD PDAC cells (Fig. S7D), thus, suggesting that budesonide regulates OXPHOS-related genes, at least in part, through the GR. Accordingly, dexamethasone showed a similar effect. In line with this idea and our findings that sublethal concentrations of OXPHOS inhibitors exerted a beneficial effect on PDAC spheroids growth (Fig. 6F), the volume of *NR3C1* KD PDAC spheroids was significantly higher compared to that of control spheroids (Fig. S7E-F).

These data support the transcriptome profile and suggest that budesonide induces a GR-dependent metabolic switch toward OXPHOS in PDAC cells, which conversely rely on glycolysis to grow in a 3D environment. We thus hypothesized that a budesonide-induced metabolic imbalance could eventually affect proliferation of PDAC spheroids. GSEA and GO analysis of DE genes in budesonide-treated spheroids (3D) revealed a significant deregulation of genes involved in cell cycle including cell cycle and mitotic spindle checkpoints (Fig. 7D-E, Fig. S7G, Tables S3-S4). Accordingly, cell cycle analysis of 3D spheroids ± budesonide showed that budesonide significantly increased the fraction of PDAC cells in G0/G1 phase, and reduced the fraction of cells in S and G2/M phases (Fig. 7F). Interestingly, among the DEGs we found the cyclin dependent kinase inhibitor 1 C (*CDKN1C*), a GR target [14] also known as p57^Kip2^, which was strongly up-regulated (∼100 fold) by budesonide in 3D spheroids. We first validated these data by qPCR analysis. *CDKN1C* expression was strongly induced by both budesonide and dexamethasone in control 3D spheroids; however, this induction was almost completely abolished in *NR3C1* KD spheroids (Fig. S7H), providing unprecedented evidence that CDKN1C is a GR target in PDAC cells. We thus asked whether the anti-proliferative effect of budesonide in 3D tumor spheroids may depend on the induction of *CDKN1C*. To test this hypothesis, we silenced *CDKN1C* in PDAC cells by siRNA (Fig. 7F). PDAC spheroids were generated using cells carrying siNT as controls or si*CDKN1C* (*CDKN1C* KD) and treated ± budesonide (1 µM) (Fig. 7G and Fig. S7I). As expected, budesonide significantly reduced the volume of control PDAC spheroids, whereas it did not affect spheroid volume in CDKN1C KD cells, suggesting that CDKN1C silencing significantly decreased the susceptibility of PDAC spheroids to budesonide-dependent growth inhibition (Fig. 7G-H).

All together these data suggest that budesonide-induced metabolic reprogramming impairs PDAC growth in 3D at least in part through induction of *CDKN1C.*

## Discussion

In this study we have investigated the effect of budesonide, a GC largely used for the treatment of chronic asthma, on PDAC, a highly aggressive form of pancreatic cancer, which is characterized by rapid progression and poor clinical outcome [1]. Clinical evidence has reported lower occurrence of PDAC in asthmatic patients [4]. Our results provide unprecedented evidence that budesonide impacts on PDAC cell behavior and that this is strictly dependent on the cell environment.

PDAC cells cultured on 2D plastic substrates generate strong cell-substrate interactions and display mesenchymal/motile features. Under such conditions, budesonide promotes cell-cell adhesive interactions and inhibits the mesenchymal/motile characteristics of PDAC cells, without affecting cell proliferation and apoptosis. These phenotypic and molecular changes are observed only in a high (µM) concentration range of budesonide and not with other GCs like dexamethasone, and are in line with results previously reported in mouse ESCs and in breast cancer cells [11–13]. However, when tested in more physiological 3D conditions, budesonide exerts a dose-dependent anti-proliferative effect on PDAC spheroids, which relies on the GR. Most remarkably, PDAC spheroids become highly susceptible to budesonide, which is effective at a nM concentration range. These results describe, for the first time to our knowledge, a growth condition-dependent anti-proliferative effect of budesonide on cancer cells. Indeed, to date only few studies have described to our knowledge the effect of budesonide on cancer cell proliferation. Specifically, it has been shown that budesonide promotes the growth of bladder cancer cells, while it reduces TP53 wild type (A549) but not mutant lung cancer cell proliferation [22, 23].

The efficacy of budesonide in reducing tumor growth by decreasing cell proliferation was confirmed in PDAC xenografts in vivo, reinforcing the idea that cancer 3D spheroids more accurately recapitulate different aspects of tumor architecture and biology [24–27]. Interestingly, the inhibitory effect of budesonide on PDAC tumor growth was similar to that of gemcitabine, a deoxycytidine analog, which inhibits DNA synthesis and is considered the gold-standard treatment for PDAC [28].

It is known that cells cultured in 2D and 3D respond differently to drug treatments. This is mainly due to differences in the physical and structural properties of the cells, as well as in cellular shape, pH, and metabolism [25]. Of note, most cancer cells are more resistant to anti-proliferative agents (mainly chemotherapeutic drugs) in 3D compared to 2D cultures, most likely due to reduced access of the drugs to the internal cell layers of the 3D tumor-like structures [24]. Conversely, here we demonstrate that budesonide exerts a potent anti-proliferative effect only on 3D spheroids. To our knowledge, no other drugs have been reported so far with a similar behavior, except for the KRAS inhibitor MRTX1133, which is more effective in inhibiting PDAC cell proliferation in 3D vs. 2D cultures [29]. However, MRTX1133 affects only a subset of PDAC cell lines, which carry a specific oncogenic mutation (G12D) in KRAS.

Genetic and pharmacological evidence indicates that budesonide differently modifies PDAC cell behavior under 2D or 3D culture conditions and that this occurs through GR-independent or GR-dependent mechanisms, respectively. So far, the role of the GR in the development and progression of solid tumors has not been completely dissected, and is still controversial. Indeed, different studies have led to the idea of a dichotomy of the GR action, which can be either tumor suppressor or tumor promoter across different types of cancer. Notably, this duality also extends to different subtypes of the same tumor, as shown in breast cancer [30]. In the case of pancreatic cancer, our results and evidence from the literature suggest a tumor-suppressive effect of GCs and the GR [30]. For instance, patients with PC who received dexamethasone during operation show increased survival rate [31, 32]. Moreover, GCs reduced proliferation and invasiveness of different PC cell lines, suggesting a tumor-suppressive effect [33, 34]. Our results support and extend these findings, providing unprecedented evidence that the environmental conditions influence the susceptibility of PDAC cells to the anti-proliferative effect of budesonide and other GCs. We suggest that this depends on the metabolic status of the cells. Several lines of evidence support this hypothesis. Genome wide transcriptome analysis suggests that PDAC cells, when cultured under 3D conditions, undergo a general metabolic reprogramming that includes protein, lipid, and energy metabolism. In this context, we demonstrate that PDAC cells growing as 3D spheroids are highly sensitive to the glycolysis inhibitor 2-DG, while treatment with sublethal concentrations of OXPHOS inhibitors, such as rotenone and metformin, increases PDAC growth. This led us to hypothesize that OXPHOS activity limits the growth of PDAC spheroids, and that budesonide reprograms PDAC cells towards a metabolically less aggressive phenotype.

To date, only few and recent studies have systematically investigated the differences in the metabolism between 2D and 3D culture settings. Interestingly, it has been recently shown that both lung and breast cancer cell lines in 3D culture conditions undergo a metabolic reprogramming with increased expression of glycolytic enzymes and an enhanced production of lactate [35]. Furthermore, metabolic flux analysis of 3D spheroids of colorectal cancer and PDAC cell lines showed that glucose and OXPHOS metabolism differ from that of 2D cultures [27]. Our results support and extend these findings providing molecular and functional evidence that PDAC 3D cultures undergo a global metabolic reprograming, which includes protein, lipid, and energy metabolism and highlight that these metabolic differences should be taken into considerations when assessing the response of PDAC cells to drugs.

It is well-known that PDAC cells are characterized by an extensive metabolic plasticity/heterogeneity [36]. For instance, while PC is considered a highly glycolytic cancer [37], tumor spheres of PDAC cancer stem cell (CSC) strongly rely on mitochondrial OXPHOS and that mitochondrial inhibition suppresses their growth, inducing apoptosis [38–40]. This apparent discrepancy with our findings is most likely due to the different culture conditions and experimental settings. Specifically, while CSCs enriched tumor spheres are cultured in serum-free medium supplemented with fibroblast growth factor (i.e., DMEM-F12/ B27 plus bFGF), the floating spheroids of PDAC cells described in this study are cultured in serum-containing medium (RPMI +10% FBS) without addition of any specific cytokines/growth factors. This is particularly relevant for our study, as it allows us to rule out the possibility that different culture conditions may affect the metabolic status of PDAC cells in 2D and 3D.

Our transcriptome data indicate that budesonide induces OXPHOS gene expression in both 2D and 3D cultures, suggesting that it similarly modifies the energy metabolism of PDAC cells in the two culture conditions. Of note, this metabolic shift towards OXPHOS contrasts with the highly glycolytic dependency of PDAC spheroids. This brings up the hypothesis that budesonide may induce a metabolic imbalance in PDAC spheroids, which limits their metabolic plasticity and results in reduced proliferation. In line with this idea, budesonide significantly modified the expression of a large set of genes associated to cell cycle/proliferation in PDAC spheroids but not in 2D culture. Mechanistically, we suggest that this effect is mediated, at least in part, by the tumor suppressor CDKN1C/p57Kip2, which is strongly induced by budesonide in a GR-dependent manner. Of note, it has been recently reported that CDKN1C induction blocks cell cycle progression of lung cancer cells [41]. Most remarkably, CDKN1C has been reported as one of the most under-expressed genes at both the RNA and protein level, in a set of intraductal papillary mucinous neoplasm (IPMN) of the pancreas, and in several pancreatic cancer cell lines [42]. These data support our findings and the role of CDKN1C as part of the mechanism underlying budesonide-dependent inhibition of PDAC spheroid growth.

Based on our findings and data from the literature, we hypothesize that budesonide can be used both in chemoprevention therapy and as a potential adjuvant drug by exploiting its different mechanisms of action on PDAC. First, besides the epidemiological study, which reported that asthmatic patients that are usually under budesonide treatment [6, 7] showed a reduction of PDAC incidence [4], a randomized double-blind trial revealed that inhaled budesonide reduces lung carcinogenesis in a population of high-risk volunteers [43, 44]. Although the cellular/molecular mechanism(s) of budesonide-dependent chemoprevention are still unknown, it has been hypothesized that inhaled GCs can prevent/reduce the transmigration of tumor-promoting immune cells into the precancerous lung lesions/nodules, delaying their transformation into lung cancer [45]. Accordingly, budesonide significantly modified the expression of different gene sets associated with pro-inflammatory and pro-fibrotic mediators in PDAC cells. Moreover, genes related to elastic fibers, ECM and matrisome, which play a critical role in PDAC development [46], are negatively enriched in budesonide-treated PDAC cells. These results are in line with our recent findings that budesonide is able to reduce collagen synthesis/accumulation both in lung and in breast cancer cells [13].

Thus, we suggest that budesonide could reduce/prevent the transformation of precancerous pancreatic lesions by (i) regulating the synthesis of inflammatory chemokines/cytokines in PDAC cells, which in turn, control the migration of immune cells into inflamed tissues, (ii) inhibiting collagen synthesis/deposition. In addition, the anti-proliferative effect of budesonide on PDAC tumor growth in vivo makes its use promising as a adjuvant therapy. In this respect, it is important to consider that the anti-proliferative effect of budesonide is GR-dependent and could be efficiently mirrored by other GCs. Conversely, budesonide, but not the classical GC dexamethasone, promotes epithelialization and reduces PDAC cell migration and invasion in a GR-independent manner, suggesting that it can also prevent/reduce the metastatic progression of PDAC. Thus, the unique ability of budesonide to reduce both PDAC cell proliferation and their mesenchymal/invasive features supports the idea that budesonide could be used in place of other GCs, like dexamethasone, in an adjuvant therapy for PDAC to prevent both tumor growth and dissemination.

In conclusion, here we provide unprecedented evidence that the transition from 2D to 3D culture sensitizes PDAC cells to GCs, and adds to the emerging evidence that the response and susceptibility of tumor cells to drugs are affected by the culture environment. Future studies will be necessary to broaden the role of the 3D environment and OXPHOS-to-glycolytic transition in the susceptibility to the GCs in other cancers. Finally, our data highlight the importance of using 3D culture conditions for drug screening applications aimed at identifying anticancer drugs targeting metabolic pathways.

### Electronic supplementary material

Below is the link to the electronic supplementary material.

Supplementary Material 1

Supplementary Material 2

Supplementary Material 3

## Data Availability

The datasets generated and/or analyzed during the current study are available in the GEO repository (submission ID: GSE255746 and GSE255747).

## Abbreviations

2-DG: 2-deoxyglucose
ALDOA: Aldolase, fructose-bisphosphate A
ATP: Adenosine triphosphate
BSA: Bovine serum albumin
Bude: Budesonide
cCas3: Cleaved caspase-3
CDKN1C: Cyclin dependent kinase inhibitor 1 C
COX: Cytochrome C oxidase
Cy3: Cyanine3
DAB: 3,3-diaminobenzidine
DAPI: 4,6-diamidine-2-phenylindole
DE: Deregulated
Dexa: Dexamethasone
DMSO: Dimethyl sulfoxide
ECL: Enhanced chemiluminescence
ECM: Extracellular matrix
EDTA: Ethylenediaminetetraacetic acid
EdU: 5-ethynyl-2’-deoxyuridine
ENO1: Enolase 1
ESCs: Embryonic stem cells
FACS: Fluorescence-activated cell sorting
FBS: Fetal bovine serum
FFPE: Formalin-fixed, paraffin-embedded
FITC: Fluorescein isothiocyanate
FN1: Fibronectin 1
GAPDH: Glyceraldehyde-3-phosphate dehydrogenase
GCs: Glucocorticoids
Gem: Gemcitabine
GO: Gene ontology
GR: Glucocorticoid receptor
GSEA: Gene set enrichment analysis
HK2: Hexokinase 2
HRP: Horseradish peroxidase
Hydro: Hydrocortisone
KD: Knock down
NDUF: NADH: ubiquinone oxidoreductase
NR3C1: Nuclear receptor subfamily 3 group C member 1
OCT: Optimal cutting temperature
OXPHOS: Oxidative phosphorylation
PBS: Phosphate buffered saline
PBSFT: Phosphate buffered saline fetal bovine serum triton
PC: Pancreatic cancer
PCA: Principal component analysis
PDAC: Pancreatic ductal adenocarcinoma
PFA: Paraformaldehyde
PFKL: Phosphofructokinase, liver type
PGAM1: Phosphoglycerate mutase 1
PI: Propidium iodide
PKH26: Lipophilic, red fluorescent dye
PKM: Pyruvate kinase M1/2
RNA-Seq: RNA sequencing
RT: Room temperature
shRNA: Short hairpin RNA
SREBP: Sterol regulatory element binding transcription
TMRE: Tetramethylrhodamine, ethyl ester
UQCR: Ubiquinol-cytochrome C reductase
VIM: Vimentin
