# Supplementary material for: Three-dimensional environment sensitizes pancreatic cancer cells to the anti-proliferative effect of budesonide by reprogramming energy metabolism
Source: J Exp Clin Cancer Res. 2024 Jun 14;43:165. doi: 10.1186/s13046-024-03072-1 (PMC11177459; doi:10.1186/s13046-024-03072-1)
Supplement: Supplementary file 3 — Supplementary Material 3 [file 13046_2024_3072_MOESM3_ESM.docx]

**Supplementary Information**

**Table of content**

Supplementary Table 1

Supplementary Table 2

Supplementary Figure 1

Supplementary Figure 2

Supplementary Figure 3

Supplementary Figure 4

Supplementary Figure 5

Supplementary Figure 6

Supplementary Figure 7

**Supplementary Table 1.** Antibodies and reagents used in this study

| **List of primary antibodies** | | |
| --- | --- | --- |
| Antibody | Source | Catalog Number |
| Fibronectin | Sigma-Aldrich | F3648 |
| Glucocorticoid Receptor | Cell signaling | 3660S |
| Vimentin | Cell signaling | #5741 |
| E-Cadherin | TakaRa | M108 |
| Ki-67 | Invitrogen | MA5-14520 |
| Gapdh | Abcam | ab8245 |
| Cleaved Caspase 3 | Cell signaling | 9664 |

| **List of secondary antibodies** | | |
| --- | --- | --- |
| Antibody | Source | Catalog Number |
| Alexa Fluor 488 Donkey anti-Rat IgG | Invitrogen | A21208 |
| Alexa Fluor 647 Donkey anti-Rabbit IgG | Invitrogen | A31573 |
| Alexa Fluor 488  Goat anti-Rabbit IgG | Invitrogen | A11008 |
| Alexa Fluor 594 Donkey anti-Rabbit IgG | Invitrogen | A21207 |
| Biotin-SP (long spacer) AffiniPure Goat Anti-Rabbit IgG, Fc fragment specific | Jackson ImmunoResearch | 111-065-046 |
| Goat Anti-Rabbit Immunoglobulins/HRP | Dako | P0448 |
| Goat Anti-Mouse Immunoglobulins/HRP | Dako | P0447 |

| **List of drugs** | | |
| --- | --- | --- |
| Name | Source | Catalog Number |
| Budesonide | Sigma-Aldrich | B7777-250MG |
| Dexamethasone | Sigma-Aldrich | D2915 |
| Hydrocortisone | Sigma-Aldrich | H0888 |
| 2-Deoxy-D-Glucose | Sigma-Aldrich | D8375 |
| Rotenone | Sigma-Aldrich | [R8875](https://www.sigmaaldrich.com/IT/it/product/sigma/r8875) |
| Gemcitabine hydrochloride | MedChemExpress | HY-B0003 |
| Metformin | Sigma-Aldrich | 317240-5GM |

| **List of reagents** | | |
| --- | --- | --- |
| Name | Source | Catalog Number |
| TMRE Assay | Abcam | ab113852 |
| Lipofectamine RNAiMAX | Invitrogen | 13778-100 |
| Silencer Select CDKN1C siRNA | Thermo Fischer | s2840 |
| Silencer Select Negative Control siRNA | Thermo Fischer | 4390843 |
| QCM Gelatin Invadopodia Assay | Millipore | ECM671 |
| Crystal violet | Euroclone | EMR029500 |
| Gelatin solution | Sigma-Aldrich | G1393 |
| Annexin V, FITC Apoptosis Detection Kit | Dojindo | AD10-10 |
| EdU Alexa Fluor 488 Flow Cytometry Assay | Thermo Fischer | C10420 |
| PKH26 | Sigma-Aldrich | MIDI26-1KT |
| Mayer′s Hematoxylin Solution | Sigma-Aldrich | MHS16-500ML |
| Eosin Y Solution, Alcoholic | Sigma-Aldrich | HT110116-500ML |
| Corning Matrigel Growth Factor Reduced Basement Membrane Matrix, LDEV-free | Corning | 354230 |
| Corning 96-well Clear Round Bottom Ultra-Low Attachment | Corning | 7007 |
| DAB Substrate Kit, Peroxidase (HRP) | Vector Laboratories | SK-4100 |
| VECTASTAIN Elite ABC-HRP Kit, Peroxidase | Vector Laboratories | PK-6100 |
| Killik O.C.T. | Bio-Optica | 05-9801 |
| Transwell | Costar | 3422 |

**Supplementary Table 2.** Primers used for qPCR

| **Gene** | **Forward primer (5´-3´)** | **Reverse primer (5´-3´)** |
| --- | --- | --- |
|  |  |  |
| ***CDKN1C*** | GCGGCGATCAAGAAGCTG | GCTTGGCGAAGAAATCGGAGA |
| ***NR3C1*** | TACCCTGCATGTACGACCAA | TCCTTCCCTCTTGACAATGG |
| ***GAPDH*** | TGCACCACCAACTGCTTAGC | TCTTCTGGGTGGCAGTGATG |
| ***PDK4*** | CGGCTTGCCAATTTCTCGTC | CCAGTCATCAGCCTCAGAGC |
| ***ATP6V1C2*** | CTTGGATTCCCTGGTTGGCC | ATGACTTCCACCACGCTCTG |


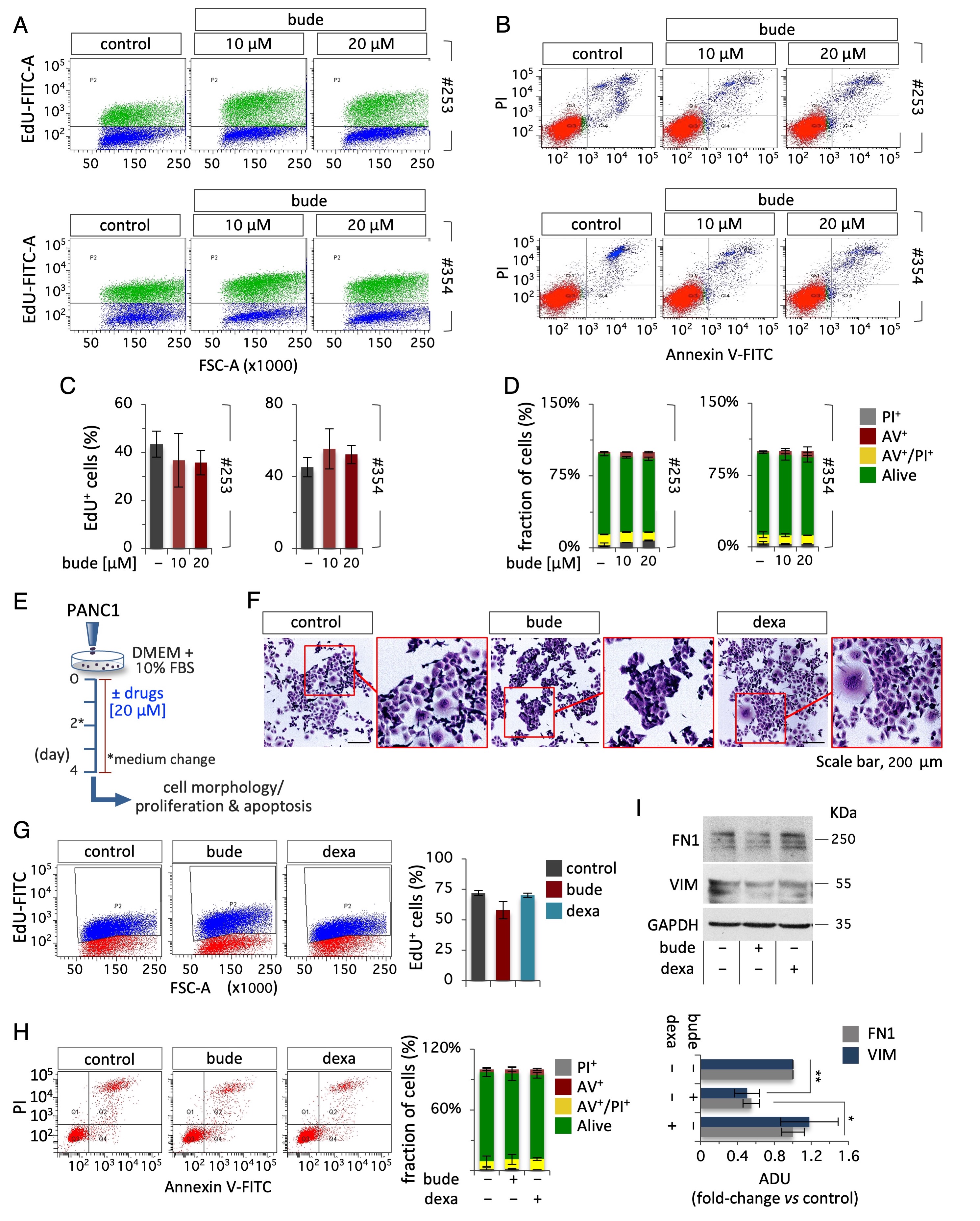


**Fig. S1**

Effect of budesonide on pancreatic cancer cell proliferation and apoptosis. **A-D** Representative flow cytometric plots (**A** and **B**) and quantification analysis (**C** and **D**) of EdU proliferation assay (**A** and **C**) and Annexin V/Propidium Iodide (PI) staining (**B** and **D**) in PDAC#253 and #354 cells ± budesonide (10 and 20 μM) or DMSO (control). The percentage (%) of EdU^+^ cells (**C**) and of Annexin V±/PI± cells are shown (**D**). **E** Schematic representation of the experimental procedure. PANC1 (5 × 10^3^ cells/cm^2^) cells were plated on gelatin-coated plates ± budesonide (20 μM), dexamethasone (20 μM) or DMSO for 4 days. The medium ± drugs was refreshed at day 2. **F** Representative crystal violet images of PANC1 cell morphology at the indicated conditions. **G** Representative flow cytometric plots of EdU incorporation in PANC1 cells treated with budesonide (20 μM), dexamethasone (20 μM) or DMSO as control (*left*) and quantification analysis of EdU^+^ cells (*right*). **H** Representative flow cytometric plots of Annexin V/PI staining in budesonide-, dexamethasone- and DMSO-treated PANC1 cells (*left*) and quantification analysis of Annexin V±/PI± cells (*right*). **I** Representative western blot analysis (*upper*) and densitometric quantification (ADU; *bottom*) of FIBRONECTIN (FN1) and VIMENTIN (VIM) in PANC1 cells treated as indicated in (**E**). ADU is shown as fold-change *vs* DMSO-treated cells, after normalization to GAPDH. Data (panels **A-D**, **G-I**) are shown as mean ± SEM (**p≤0.005; *p≤0.05; n=3, Student’s t-test).


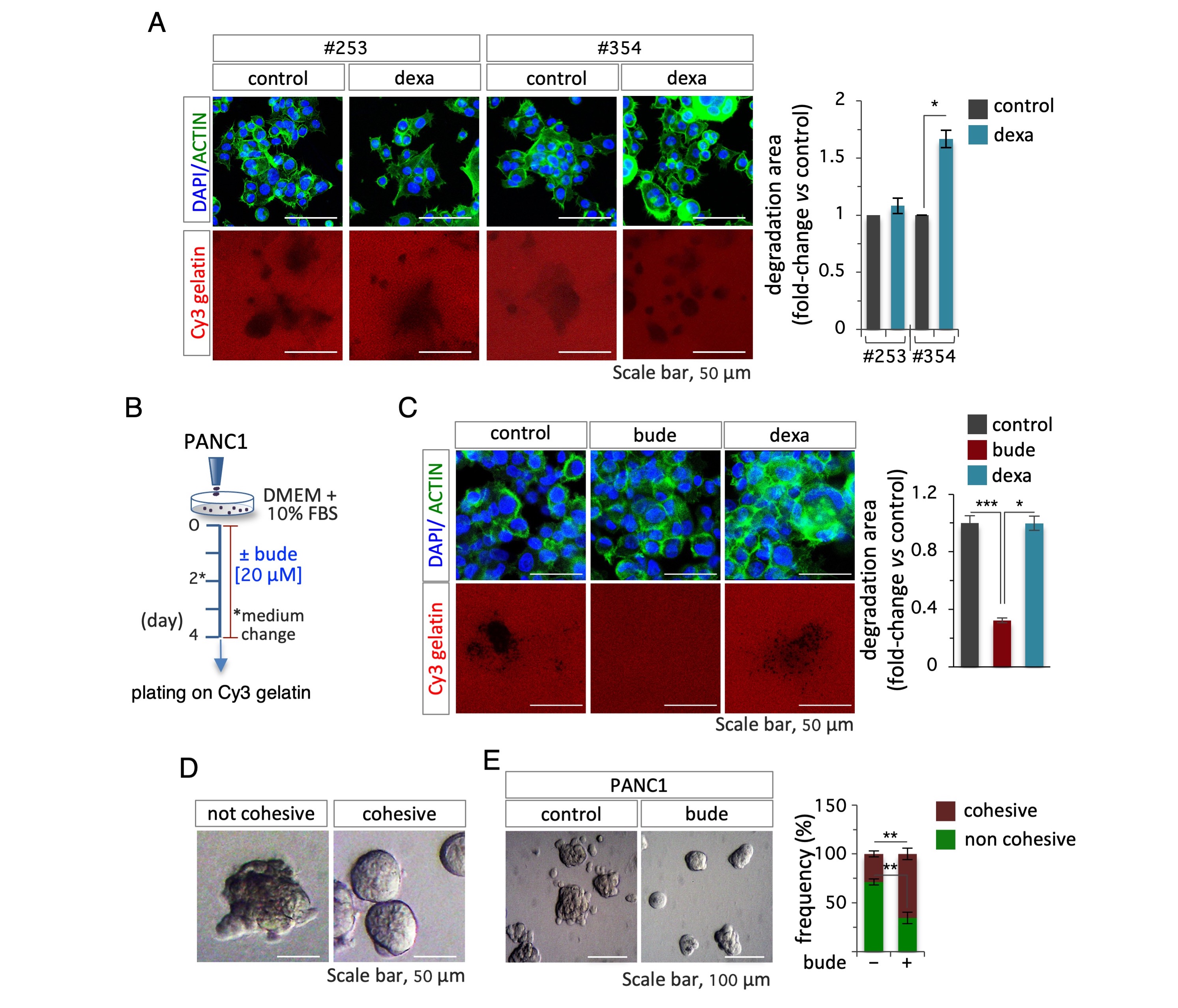


**Fig. S2**

Budesonide inhibits PDAC/PANC1 cell migration and invasion. **A** Representative confocal images (*left*) of ACTIN staining (green) in PDAC#253 and #354 cells ± dexamethasone and quantification (*right*) of Cy3-gelatin degraded area. Invasion was quantified 6 h after seeding. Nuclei were counterstained with DAPI. Data are mean ± SEM (*p<0.05; n=3, Student’s t-test) after normalization *vs* the total number of nuclei. **B** Schematic representation of the experimental procedure. Cells were plated at 1 × 10^5^ cells/cm^2^ on Cy3-conjugated gelatin and the invasion area was quantified after 72 h**. C** Representative confocal images (*left*) of ACTIN (green) staining in PANC1 cells ± budesonide (bude), ± dexamethasone and quantification (*right*) of Cy3-gelatin degraded area. Nuclei were counterstained with DAPI. Data are mean ± SEM (*p<0.05, ***p<0.001; n=3, Student’s t-test) after normalization *vs* the total number of nuclei. **D** Representative phase-contrast images of cohesive and non cohesive structures in PANC1 spheroids. **D** Representative phase-contrast images (*left*) and frequency (*right*) of cohesive *vs* non cohesive structures in PANC1 spheroids ± budesonide at day 7 after plating. DMSO was used as control. Data are mean ± SEM (***p<0.001; *p<0.05; n=4, Student’s t-test).

**
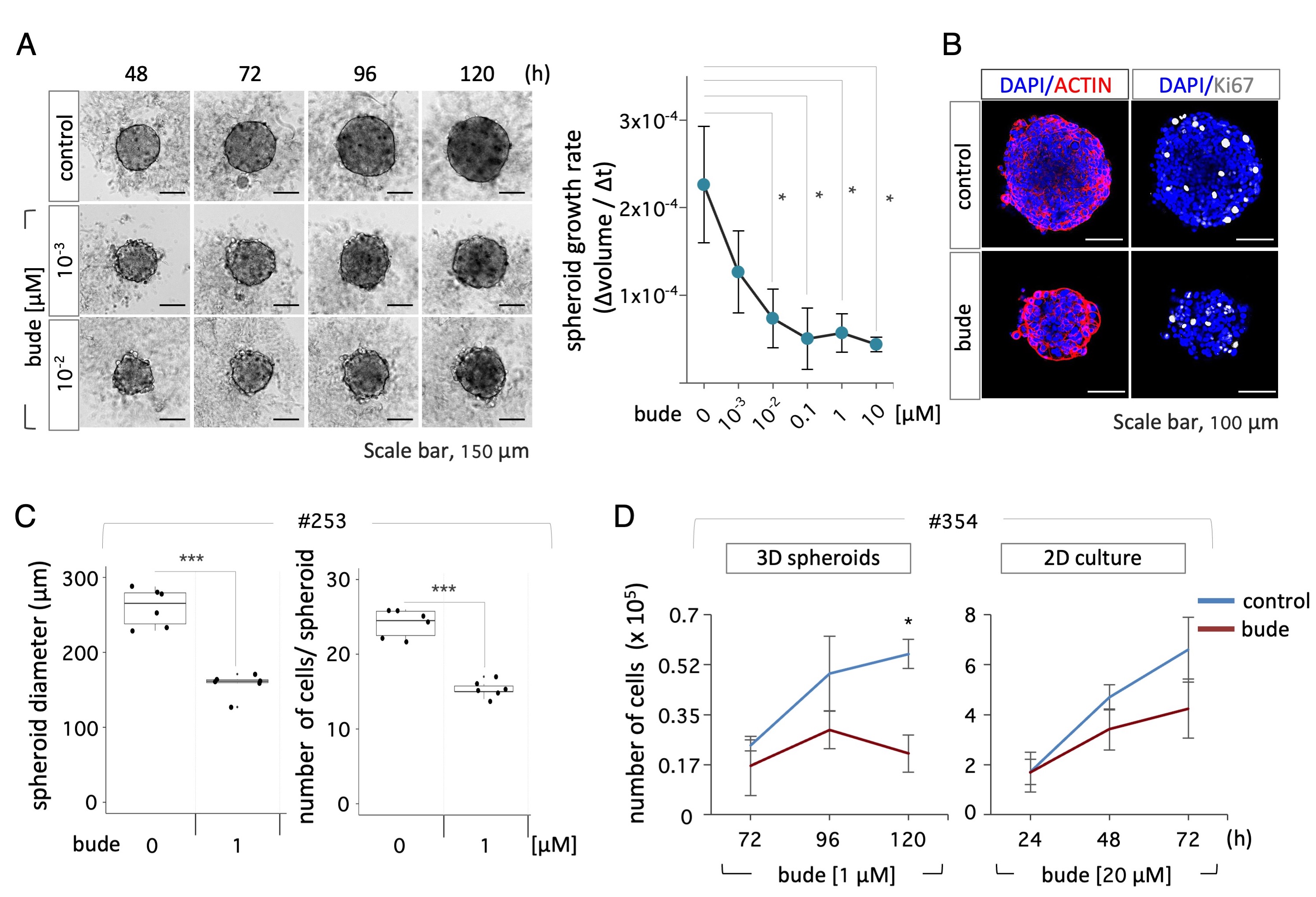
**

**Fig. S3**

Budesonide reduces the volume of pancreatic cancer spheroids. **A** Representative pictures (*left*) of PDAC#354 spheroids ± budesonide and spheroid growth rate (*right*) calculated as the mean of the ratio between the Δvolume and the Δtime (48, 72, 96 and 120 h). Data are mean ± SEM (*p<0.05; n=3, Student’s t-test). **B** Representative confocal images of ACTIN (red) and Ki67 (grey) staining in PDAC#354 spheroids ± budesonide (1 μM). Nuclei were counterstained with DAPI (blue). **C** Quantification of spheroid diameter (*left*) and number of cells/spheroid diameter (*right*) in PDAC#253 spheroids ± budesonide. The number of cells/spheroid diameter was calculated in the middle z-stack of the confocal images, using ImageJ. Data are mean ± SD (***p<0.001; n=3, Student’s t-test). **D** Time course analysis of PDAC#354 cell number in 3D spheroids ± budesonide (*left*; bude: 1 μM), and in 2D culture ± budesonide (*right*; bude: 20 μM). Data are mean ± SD (*p<0.05; n=3, Student’s t-test).


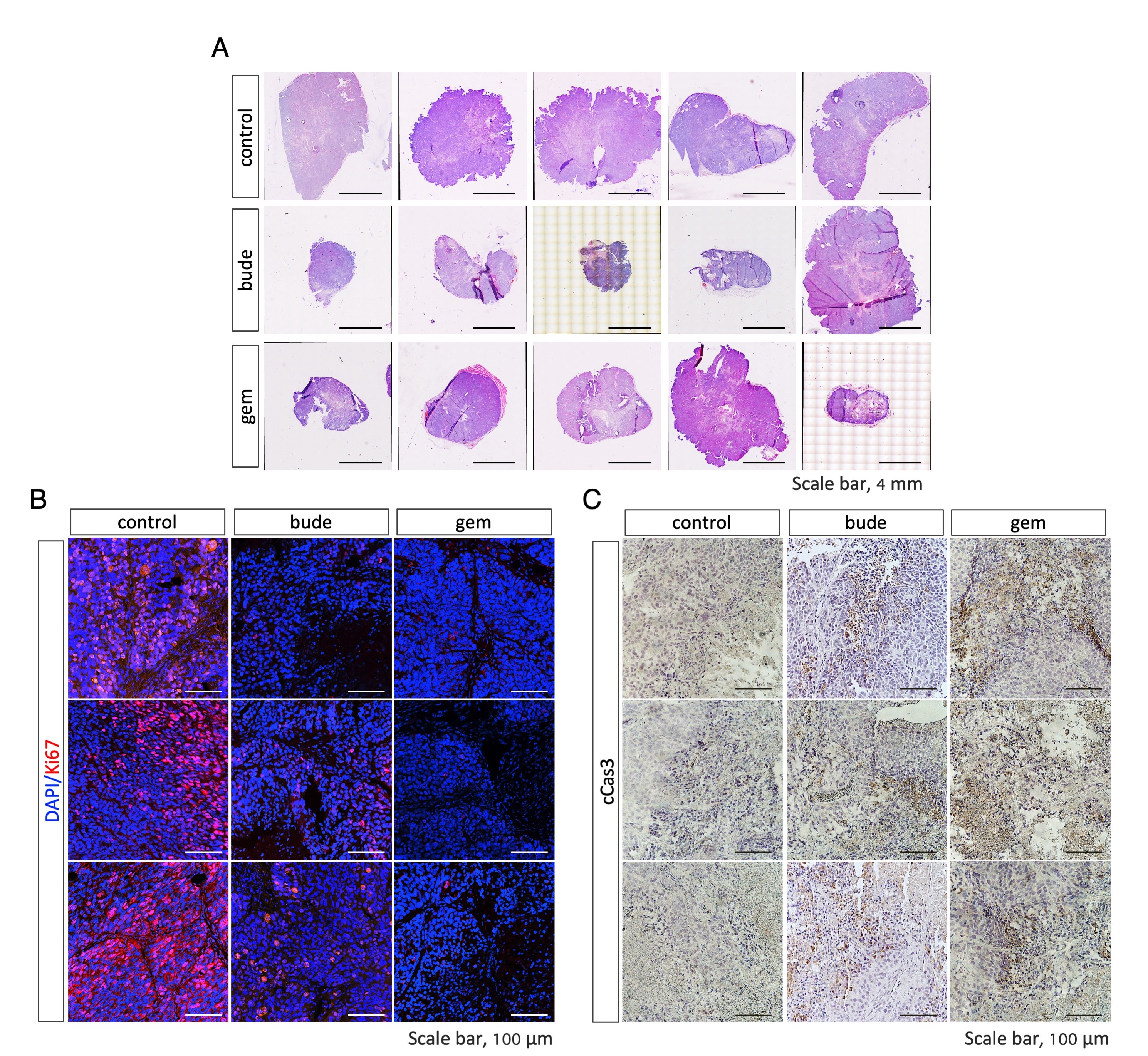


**Fig. S4**

Budesonide exerts an antiproliferative effect on PDAC tumor growth *in vivo*. **A** Representative images of mosaic reconstruction of H&E-stained tumor sections from control (vehicle), budesonide (bude)- and gemcitabine (gem)-treated mice. **B-C** Representative images of Ki67 staining (red; **B**) and cleaved Caspase3 immunohistochemistry (**C**) on tumor sections derived from budesonide (bude)-, gemcitabine (gem)-treated and control mice. Nuclei were counterstained with DAPI (blue) or hematoxylin.


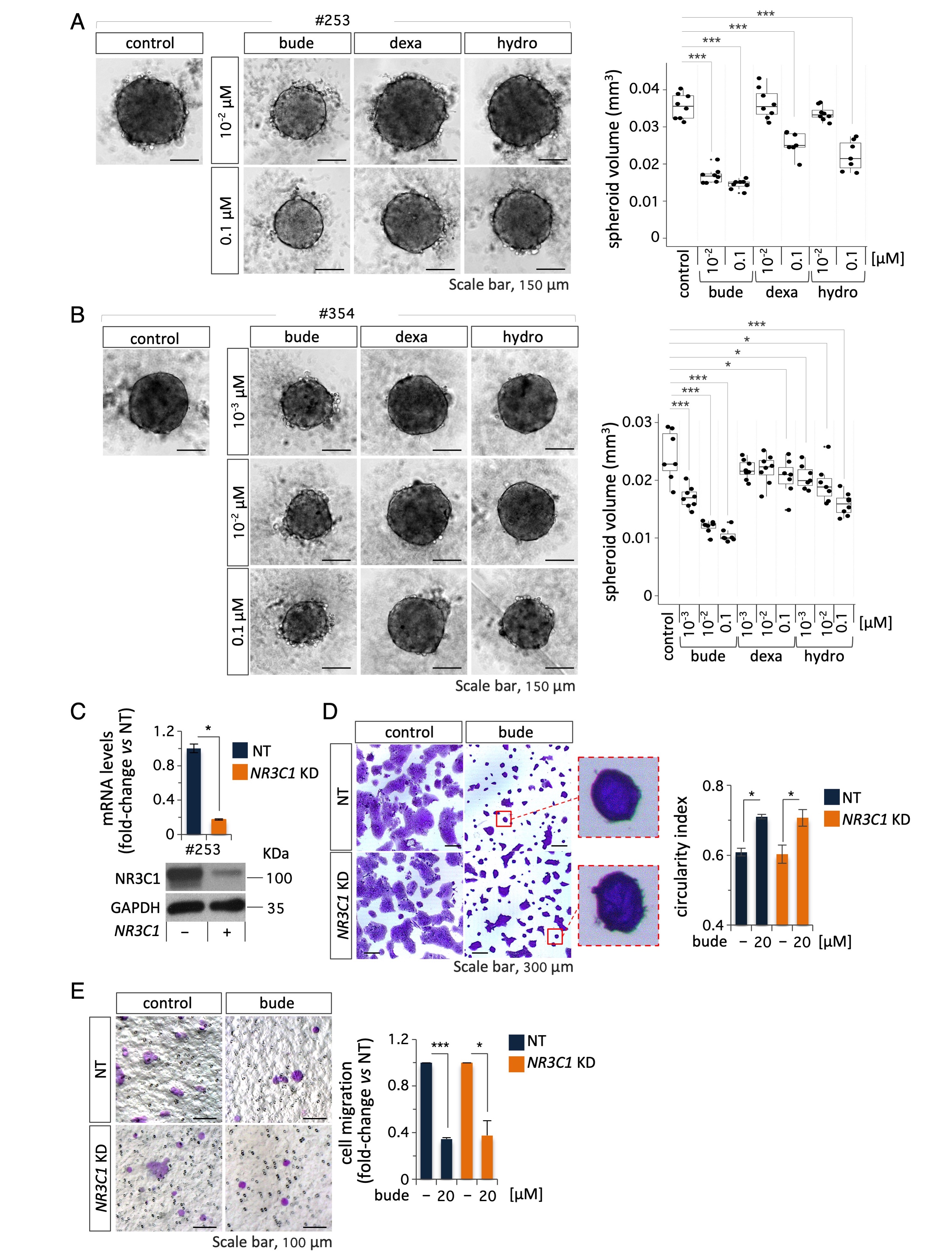


**Fig. S5**

Effects of budesonide on *NR3C1* KD PDAC cells. **A-B** Representative pictures (*left*) and volume quantification (*right*) of PDAC#253 (**A**) and #354 (**B**) spheroids ± budesonide (bude), dexamethasone (dexa), hydrocortisone (hydro) at the indicated concentrations, or DMSO as a control. Data are mean ± SD (*p<0.05; ***p<0.001; n=3, Student’s t-test). **C** qPCR (*upper*) and western blot (*bottom*) analysis of *NR3C1* expression in NT (control/ShEmpty) and *NR3C1* KD PDAC#253 cells. Data are shown as fold-change *vs* control after normalization to GAPDH and are mean ± SEM (*p<0.05; n=3, Student’s t-test). **D** Representative pictures (*left*) and circularity index quantification (*right*) of NT and *NR3C1* KD PDAC#253 cells treated ± budesonide (20 μM) and stained with crystal violet. (*p<0.05; n=3, Student’s t-test). **E** Representative crystal violet images (*left*) and quantification (*right*) of NT and *NR3C1* KD PDAC#253 cells (± budesonide 20 μM) that have migrated through the transwell. Data are shown as fold-change *vs* control and are mean ± SEM (*p<0.05; ***p<0.001; n=3, Student’s t-test).


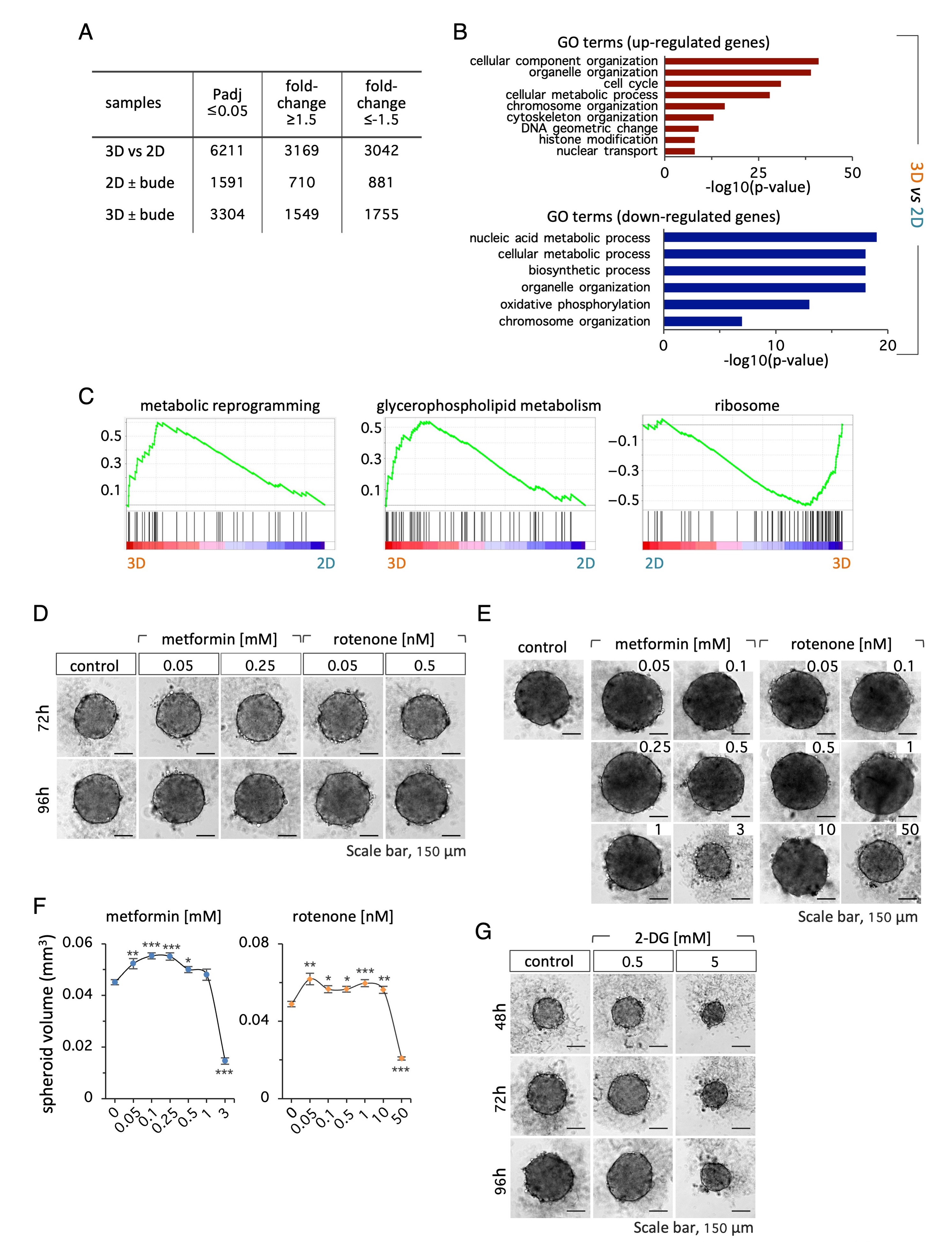


**Fig. S6**

Transcriptome profiling of PDAC cells and sensitivity to OXPHOS and glycolysis inhibitors. **A** Table showing the differentially expressed genes (DEGs) in PDAC#253 cells ± budesonide in 3D *vs* 2D cultures. The total number of DEGs (padj ≤ 0.05), the up-regulated and down-regulated genes are shown. **B** GO analysis (Biological Process) of up-regulated (*upper*) and down-regulated (*bottom*) DEGs in control PDAC 3D *vs* 2D cultures. **C** Selected GSEA plots of gene sets related to metabolic reprogramming, glycerophospholipid metabolism and ribosome in control PDAC 3D and 2D cultures. **D** Representative pictures of PDAC spheroids ± metformin (0.05 and 0.25 mM), rotenone (0.05 and 0.5 nM) or DMSO (control) at 72 and 96 h. **E-F** Dose-dependent effect of metformin and rotenone on PDAC spheroids. Representative pictures (**E**) and volume quantification (**F**) of PDAC spheroids ± metformin (0.05 mM to 3 mM), rotenone (0.05 to 50 nM) or DMSO (control) at 120 h. Data are mean ± SEM (*p<0.05; **p<0.005; ***p<0.001; n=3, Student’s t-test). **G** Representative pictures PDAC spheroids ± 2-DG (0.5 and 5 mM) at 48, 72 and 96 h. DMSO was used as control.


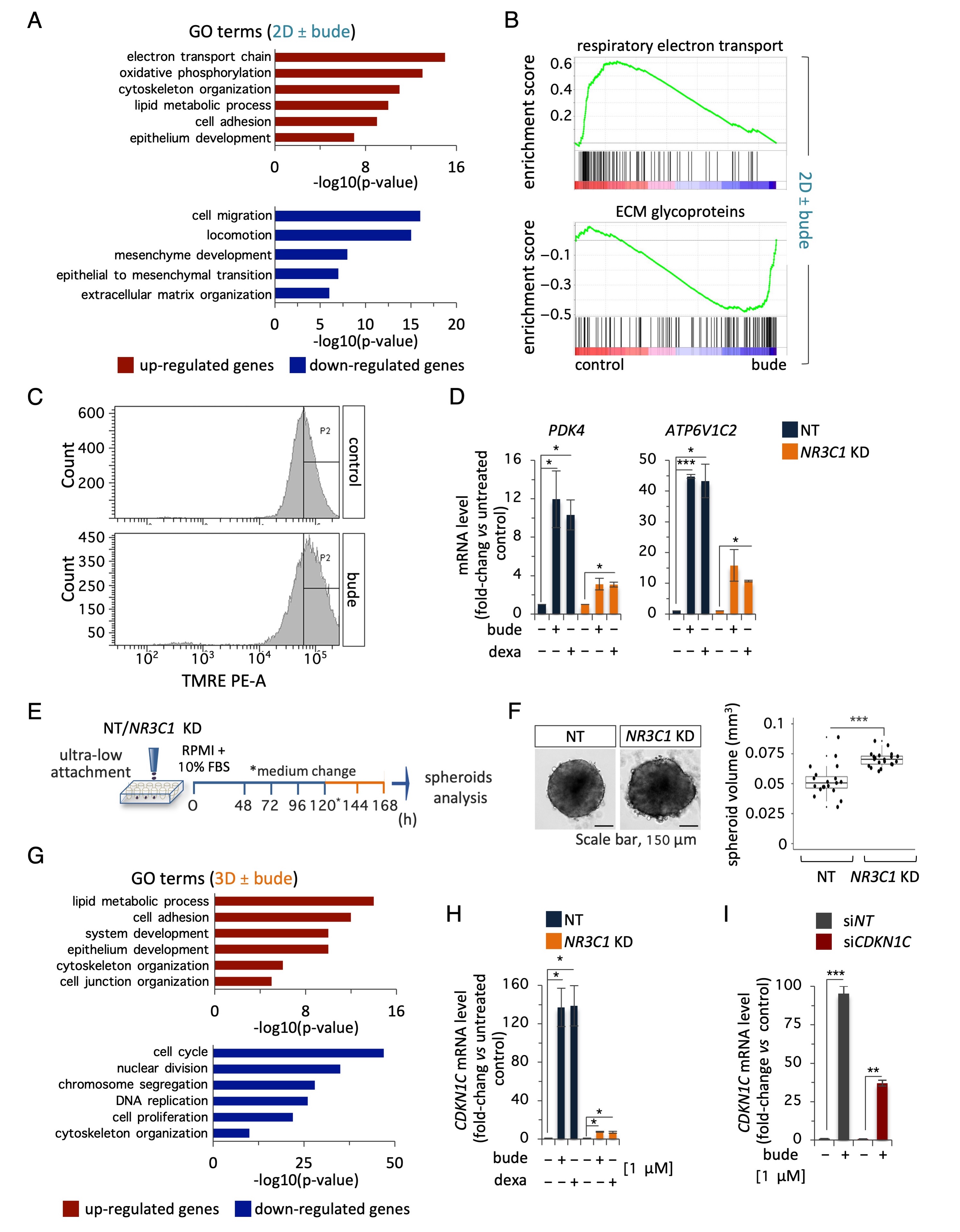


**Fig. S7**

RNA-seq analysis of PDAC cells ± budesonide in 2D and 3D cultures. **A** GO analysis (Biological Process) of up-regulated (*upper*) and down-regulated (*bottom*) DEGs between control and budesonide-treated (bude) PDAC cells in 2D cultures. **B** Selected GSEA plots of positively and negatively enriched gene sets in budesonide-treated PDAC#253 cells in 2D culture. **C** Representative FACS plots of TMRE^+^ PDAC cells ± budesonide (1μM) in 3D cultures. **D** qPCR analysis of *PDK4* and *ATP6V1C2* expression in NT (control/ShEmpty) and *NR3C1* KD PDAC#253 spheroids ± budesonide (1μM), or ± dexamethasone (1μM). Data are shown as fold-change *vs* DMSO after normalization to GAPDH and are mean ± SEM (*p<0.05; ***p<0.001; n=3, Student’s t-test). **E** Schematic representation of the experimental design. NT (control) and *NR3C1* KD PDAC#253 cells were seeded in ultra-low attachment plates (5 × 10^2^ cells/well) for 7 days. Medium was refreshed at day 5. **F** Representative pictures (*left*) and volume quantification (*right*) of spheroids generated from control and *NR3C1* KD PDAC#253 cells at 168 h. Data are mean ± SD (***p<0.001; n=3, Student’s t-test). **G** GO analysis (Biological Process) of up-regulated (*upper*) and down-regulated (*bottom*) DEGs between control and budesonide-treated (bude) cells in 3D culture. **H** qPCR analysis of *CDKN1C* expression in control (NT/ShEmpty) and *NR3C1* KD PDAC#253 spheroids ± budesonide (1 μM), or ± dexamethasone (1 μM). Data are shown as fold-change *vs* DMSO after normalization to GAPDH and are mean ± SEM (*p<0.05; n=3, Student’s t-test). **I** qPCR analysis of *CDKN1C* expression in si*CDKN1C* and siNT PDAC#253 spheroids ± budesonide (1 μM). Data are shown as fold-change *vs* DMSO after normalization to GAPDH and are mean ± SEM (**p<0.005; ***p<0.001; n=3, Student’s t-test).
